# Genome-wide CRISPR screen identified a role for commander complex mediated ITGB1 recycling in basal insulin secretion

**DOI:** 10.1016/j.molmet.2022.101541

**Published:** 2022-07-11

**Authors:** Liu Yang, Margret A. Fye, Bingyuan Yang, Zihan Tang, Yue Zhang, Sander Haigh, Brittney A. Covington, Kai Bracey, Justin W. Taraska, Irina Kaverina, Shen Qu, Wenbiao Chen

**Affiliations:** 1 Department of Endocrinology and Metabolism, Shanghai Tenth People's Hospital, School of Medicine, Tongji University, Shanghai 200072, China; 2 Department of Molecular Physiology and Biophysics, Vanderbilt University School of Medicine, Nashville, TN 37232, USA; 3 Department of Cell and Developmental Biology, Vanderbilt University School of Medicine, Nashville, TN 37232, USA; 4 Biochemistry and Biophysics Center, NHLBI, NIH, Bethesda, MD 20892, USA

**Keywords:** Commander, Integrin beta1, CRISPR screen, Insulin secretion, Endosomal recycling, Focal adhesions

## Abstract

**Objectives:**

Pancreatic beta cells secrete insulin postprandially and during fasting to maintain glucose homeostasis. Although glucose-stimulated insulin secretion (GSIS) has been extensively studied, much less is known about basal insulin secretion. Here, we performed a genome-wide CRISPR/Cas9 knockout screen to identify novel regulators of insulin secretion.

**Methods:**

To identify genes that cell autonomously regulate insulin secretion, we engineered a Cas9-expressing MIN6 subclone that permits irreversible fluorescence labeling of exocytic insulin granules. Using a fluorescence-activated cell sorting assay of exocytosis in low glucose and high glucose conditions in individual cells, we performed a genome-wide CRISPR/Cas9 knockout screen.

**Results:**

We identified several members of the COMMD family, a conserved family of proteins with central roles in intracellular membrane trafficking, as positive regulators of basal insulin secretion, but not GSIS. Mechanistically, we show that the Commander complex promotes insulin granules docking in basal state. This is mediated, at least in part, by its function in ITGB1 recycling. Defective ITGB1 recycling reduces its membrane distribution, the number of focal adhesions and cortical ELKS-containing complexes.

**Conclusions:**

We demonstrated a previously unknown function of the Commander complex in basal insulin secretion. We showed that by ITGB1 recycling, Commander complex increases cortical adhesions, which enhances the assembly of the ELKS-containing complexes. The resulting increase in the number of insulin granules near the plasma membrane strengthens basal insulin secretion.

## Highlights

• A MIN6 clone was developed for high throughput assay of insulin secretion in bulk and in single cells.
• Genome-wide CRISPR screen in the clone identified Commander complex among the top hits.
• Commd3^-/-^ cells exhibited reduced number of docked Insulin granules and exocytic events in low glucose conditions.
• ITGB1 recycling was impaired in Commd3^-/-^ cells in low glucose, causing diminished cortical focal adhesions and ELKS complexes.

## 1 Introduction

Insulin secreted from pancreatic beta cells is used to stimulate glucose uptake for cellular fuel and storage. Insulin secretion is continuous but peaks soon after meals, upon glucose stimulation [1]. Glucose is the most potent stimulus of insulin secretion. Inappropriate responses to glucose by beta cells can cause hypoglycemia and hyperglycemia, with the latter representing a hallmark of diabetes. Much has been learned about glucose-stimulated insulin secretion (GSIS) in the past 6 decades [2]. However, much is still unknown about insulin secretion, particularly during fasting [3].

A prerequisite for insulin secretion is the biogenesis of insulin granules and their trafficking to the plasma membrane [4]. Insulin granules, both newly formed in the Golgi complex and recycled from endosomes, migrate along cytoskeletal tracks to the plasma membrane. The cortical microtubule stabilizing center (CMSC) that anchors microtubules is critical for the delivery of insulin granules to the proximity of the plasma membrane [5]. Upon arrival, insulin granules dock on the plasma membrane by the clustering syntaxin1 and Munc18-1 family [6]. Docking is critical for granules to be secreted following priming and a rise of intracellular Ca^2+^, which triggers the fusion of the plasma membrane and the granule membrane [7].

Identifying the mechanism of glucose-stimulated insulin secretion (GSIS) has been a major research focus [7]. Products from glucose metabolism induce insulin secretion through at least two pathways: the ATP-mediated triggering pathway and other metabolite-mediated amplifying pathway. The triggering pathway has been extensively described [7]. It consists of the ATP-sensitive K+ channel and voltage-dependent Ca2+ channels. In contrast, the amplifying pathway is not well defined. Several glucose metabolites have been proposed to activate the amplifying pathway, including acetyl-CoA [8], phosphoenolpyruvate [9], NADPH [10], and adenylosuccinate [11]. However, the mechanism by which they amplify GSIS is not clear [12].

Half of the daily insulin secreted is released during fasting when blood glucose is at basal levels. Basal insulin is important for maintaining fasting blood glucose by inhibiting hepatic gluconeogenesis and glycogenolysis. Regulation of basal insulin secretion is not well studied compared to GSIS [3]. Similar to asynchronous release and spontaneous release of synaptic vesicles, basal insulin secretion may involve multiple mechanisms [13]. The cellular composition of synaptotagmins (Syt) with different Ca^2+^ sensitivity can influence basal insulin secretion. For example, the high basal insulin secretion of immature beta cells is a result of higher Ca^2+^ sensitivity due to lower expression of Ca^2+^-insensitive Syt4 [14]. However, a dynamic change of Ca^2+^ sensitivity in adult beta cells has not been reported. The number of docked insulin granules also regulate basal insulin secretion. For example, loss of Rim2α, a protein important for insulin granule docking, leads to lower basal insulin secretion [15]. Other mechanisms that regulate basal insulin secretion remain to be discovered.

Integrin β1 (ITGB1) plays an important role in beta cell development and function. Constitutive loss of *Itgb1* in beta cells impairs their proliferation [16], while conditional loss of *Itgb1* in beta cells impairs GSIS [17]. ITGB1 targets insulin granules to the vascular facing cell cortex through focal adhesion-mediated positioning of the presynaptic scaffold proteins ELKS and Liprin [18,19]. ELKS is a multivalent protein that can interact with multiple components of the secretory machinery, including Liprin-α isoforms, RIMs, bassoon, LL5β and voltage-dependent Ca2+ channels (VDCC) [20]. In pancreatic beta cells, ELKS is localized at the secretion sites of insulin granules, consistent with its scaffolding role in the secretion sites [21]. Although ELKS is important for synaptic vesicle docking in neurons [22], loss of ELKS in beta cells does not change insulin granule docking [23], possibly due to functional redundancy [22]. ELKS increases VDCC levels in vascular adjacent membrane [23]. Through its interaction with LL5β, ELKS can direct insulin granule distribution by anchoring the cortical microtubule stabilizing complex (CMSC) to focal adhesions [5].

Genome-wide CRISPR screen is a promising approach to better understand insulin secretion [24]. The CRISPR system consists of a Cas9 protein and a guide RNA, often in the form of single guide RNA (sgRNA). Each sgRNA targets Cas9 to a specific sequence. In a genome-scale screen, a library of sgRNAs (usually consists of 100,000 sgRNAs) targeting all genes are expressed individually with Cas9 to generate a library of mutant cells. Cells with abnormalities are collected to determine the underlying causes. The sgRNAs that are enriched or depleted in these cells identify the responsible genes. For functional defects that do not change viability like GSIS, however, a high throughput method to identify individual cells with functional defects is necessary for such screens. Currently, no high-throughput single cell secretion assay in beta cells is available. The lack of such an assay for insulin secretion has prevented the application of Cas9-based screen in insulin secretion. Therefore, we developed a system for assaying single cell secretion in beta cells and performed a genome-wide CRISPR screen to identify candidate genes that, when mutated, increase or decrease the ratio of secretion at high and low glucose concentrations. We identified a role for the Commander complex in insulin granule docking and insulin secretion under basal glucose conditions. Mechanistically, the Commander complex promotes basal insulin secretion at least in part through recycling ITGB1 from Rab4-positive endosomes to the plasma membrane. Defective ITGB1 recycling decreases the number of cortical focal adhesions and the ELKS-containing complexes. Defective ITGB1 recycling also reduces the number of docked insulin granules and their secretion.

## 2 Materials and methods

### 2.1 Antibodies, plasmids and other reagents

Primary antibodies diluted in PBS with 1% BSA (for immunofluorescence) or 5% BSA (for immunoblotting) were Cas9 (Cell Signaling Technology, #14697), COMMD1 (Proteintech, 11938-1-AP), COMMD3 (Proteintech, 26240-1-AP), COMMD5 (Proteintech, 10393-1-AP), CCDC22 (Proteintech, 16636-1-AP), CCDC93 (Proteintech, 20861-1-AP), ITGB1 (Millipore, MAB1997), ITGAV (BD Biosciences, 550,024), Vincullin (Santa Cruz, sc-73614), Paxillin (BD Biosciences, 610,051), and ELKS (Abcam, ab50312). Phalloidin was from Invitrogen (A22287). Plasmid pLenti-Cas9-Blasticidin and plentiCRISPR-E (Addgene #78852) were gifts from Feng Zhang (MIT and Broad Institute) (Addgene #52962). EGFP tagged Canine Rab4, Rab5, Rab7, and Rab11 were gifts from Marino Zerial (Max Planck). A plasmid containing the coding sequence of SNAP-PHOGRIN-EGFP was constructed by HiFi assmebly using PCR fragments. The final reporter construct was made by HiFi Assembly using PCR fragments containing SNAP-PHOGRIN-EGFP, PROINUSLIN-NanoLuc (a gift from David AItshuler, addgene #62057), and pLenti-RIP (a gift from David Jacobson, Vanderbilt). The mutant ITGB1 was made by linear amplification of pcDNA3.1-hITGB1 (a gift from Roy Zent, Vanderbilt Medical Center) using Q5® High-Fidelity DNA Polymerase and two primers: 5′-GGACACGGGTGAAGCTGCTATTTATAAGAGTGCC-3′ and 5′-TAACAACTGTGGTCGCTGCGAAGTATGAGGGA-3’.

SNAP substrates were purchased from NEB (SNAP-Surface® Block, S9143S; SNAP-Surface® Alexa Fluor® 546, S9132S; SNAP-Surface® Alexa Fluor® 647, S9136S).

### 2.2 Lentivirus production

HEK293FT cells (a gift frorm David Jacobson lab, Vanderbilt) were used for virus packaging. Cells were cultured in DMEM GlutaMax with 10% FBS, 100IU/mL penicillin, and 100 mg/mL streptomycin to 50–70% confluency in 100 mm dishes. The media was then switched to DMEM GlutaMax with 10% FBS for transfection. The following DNA mixture (18.65 μg lentiviral plasmid, 13.9 μg packaging plasmid (pCMV-dR7.74psPAX2), and 5.56 μg envelope plasmid (pMD2. G) was used together with Takara CalPhos™ Mammalian Transfection Kit (#631312) as instructed. Cells were incubated with the DNA mixture for 5–7 h and then the transfection media was aspirated and replaced with fresh DMEM GlutaMax with 10% FBS. Medium containing lentivirus was harvested 72 h later and frozen at −80 °C. The viral medium was thawed for transduction of MIN6 cells.

### 2.3 Cell culture, viral transduction and selection

MIN6 cells were obtained from Addexbio and grown in AddexBio Advanced DMEM Medium(C0003-04) containing 15% FBS, 0.05 mM 2-mercaptoethanol, and penicillin (100 U/ml)/streptomycin (100 μg/mL). Cells were cultured at 37 °C with 5%CO_2_. The cells were transduced at seeding to enhance the efficiency. Polybrene (10 μg/mL) was added with 3rd-generation lentiviruses containing the above-mentioned lentiviral plasmids. The virus was left on the cells for 24 h and then replaced with fresh media. The virus was then allowed to express for 2–3 days. After transduction, reporter expression was confirmed by equivalent EGFP expression and Cas9 expression was confirmed by western blot.

### 2.4 Glucose-stimulated insulin secretion assays (NanoLuc and ELISA)

The MIN6 cells were seeded in a 12-well plate to 80–90% confluency. Cells were starved for 1 h in 1 mL KRB (114 mM NaCl, 5 mM KCl, 1.2 mM MgSO4, 1.2 mM KH2PO4, 20 mM HEPES, 2.5 mM CaCl2, 25.5 mM NaHCO3 and 0.2% BSA) containing 1 mM glucose. They were then cultured with freshly prepared KRB with 1 or 25 mM glucose for another hour. In glucose response curve experiment, cells were cultured in freshly prepared 1 mM, 5.6 mM, 11 mM and 25 mM KRB for another hour after starvation. The conditioned buffer was collected and centrifuge at 2,000 rpm for 5 min to obtain supernatants. The cells were lysed with RIPA buffer (R0278, Sigma) followed by centrifugation to obtain supernatants. The supernatants were then measured for NanoLuc using Promega Nano-Glo® Luciferase Assay System (Promega) or for insulin using a mouse insulin ELISA kit (Mercodia), and DNA using AccuBlue® dsDNA Quantitation kit (Biotium) following manufacturer's instruction. The reactions were read using a BioTek Synergy™ H4 Hybrid Microplate Reader. NanoLuc and Insulin content was normalized to total DNA content (AccuBlue® dsDNA Quantitation, Biotium) in each sample. At least three independently generated sets of samples were analyzed under each condition.

### 2.5 SNAP staining of MIN6-6 cells in GSIS, imaging and flow cytometry analysis

MIN6-6 cells were starved for 4 h in DMEM with 10% FBS and 1 mM glucose followed by a 15-minute treatment with SNAP-Surface® Block (5 μM) in the same medium. After washing out the blocker, cells were sequentially treated with DMEM with 10% FBS, 1 mM glucose and 5 μM SNAP-Surface® Alexa Fluor® 546 for 20 min and DMEM with 10% FBS, 25 mM glucose and 5 μM SNAP-Surface® Alexa Fluor® 647 for another 20 min, with washes after each treatment. For imaging, cells were pre-seeded on coverslips. After staining, cells were fixed in 4% PFA, mounted on slides with AquaMount (Thermo Scientific), and imaged with Zeiss LSM880 laser scanning spectral confocal microscope (Carl Zeiss). For flow cytometry, a positive control group was treated with 40 mM KCl instead of 25 mM glucose, and a negative control group was treated with 300uM Diazoxide in the presence of 25 mM glucose. Cells were harvested and washed before fixation in 4% PFA. Fluorescence intensity of Alexa Fluor 647 and Alexa Fluor 546 was determined using a 5-laser BD LSRII, and data were analyzed using FlowJo software (BD Biosciences).

### 2.6 Genome-wide CRISPR screens

Mouse CRISPR Knockout Pooled Library (Brie) [25] was purchased from Addgene. Approximately 150 million MIN6-6 cells were transduced at low multiplicity of infection (0.3–0.4). Transduced cells were selected and expanded in puromycin-supplemented media over 14 d to allow time for gene-editing and depletion of the target proteins before experiments. Two independent screens were performed. For each screen, 300 million cells were starved, blocked and sequentially stained with SNAP Alexa Fluor 546 and 647 in 1 mM and 25 mM glucose medium, respectively as described above. The cells were then washed twice in cold PBS, dissociated by 0.25% Trypsin with EDTA (Gibco), and fixed in 4% PFA-PBS for 15 min at room temperature with intermittently vortexing. Cells were then washed twice with cold PBS and resuspend in PBS +1% BSA. The cells were FACS sorted into the top 5% or bottome 5% according to their Alexa Fluor 647/Alexa Fluor 546 ratio using a 5-laser BD FACSAria III. Approximately 10 million cells were recovered for each population. Sorted cells were then pelleted by centrifugation, and the cell pellet was frozen at −80 °C before genomic DNA isolation. Approximately 80 million unsorted cells (1,000 × coverage per library element) were saved for screen data normalization. Genomic DNA was extracted using NK cell lysis buffe (50 mM Tris–HCL, 50 mM EDTA, 1% SDS, pH = 8.0) from sorted or unsorted cells, respectively. The genomic DNA were sent to CD Genomics for sequencing of sgRNAs. Readcount tables and gene enrichmentanalysis were performed using the MAGeCK algorithm (https://sourceforge.net/projects/mageck/).

### 2.7 CRISPR-Cas9 genome editing of candidate genes

To edit *Commd3* and *Commd5*, two independent guide sequences were selected targeting the 5′ constitutive exons of the gene. The sequence of the two *Commd3* sgRNA are: 5′-GAGAGCCGCTTACCTAGCG-3′, 5′-GTTCGCGCTTCTCCTCCGGG-3’. The sequence of the two *Commd5* sgRNA are: 5′-ATTCGACACACGAGGCAGCG-3′, 5′-CGCTGCCTCGTGTGTCGAAT-3’. Oligonucleotides containing the guide sequence were cloned into the pLentiCRISPR-E vector (# 78,852; Addgene). Lentiviruses produced from the CRISPR plasmids were used to transduce MIN6 or MIN6-6 cells as mentioned above. Cells transduced with virus from empty pLentiCRISPR-E vector were used as the control. The transduced cells were consecutively selected using 1 μg/mL puromycin for 7 days (P8833; Sigma). Gene editing was confirmed by western blot.

### 2.8 Immunoblotting

Cells were lysed in RIPA lysis buffer (R0278, Sigma) supplemented with a protease inhibtor (30,496,700, Roche) and phosphatase inhibitor cocktail (P5726, Sigma). Lysates were boiled in 1 × sample buffer (#1610747, Bio-Rad), resolved on 4–20% Mini Protean TGX Precast Gel (#4561096, Bio-Rad), and transferred onto PVDF membranes (IPFL00010, Millipore) using a Bio-Rad Trans-Blot system. Membranes were blocked with 5% BSA in TBS with Tween-20 for 60 min at RT. Primary antibodies were diluted in blocking buffer and incubated overnight at 4 °C. HRP-conjugated secondary antibodies (Bio-Rad Laboratories or Abcam) diluted in blocking buffer at 1:2,500 were incubated for 2h at RT and developed using ECL (WBKLS0100, Miliipore). Blots were imaged using an ChemiDoc MP Imaging System (Bio-rad) and quantified using ImageJ software.

### 2.9 Real-time qPCR

*Commd3* knock down and control cells were washed two times with cold PBS prior to lysis by TRIZOL reagent. RNA was isolated using Direct-Zol columns and concentrated using RNA Clean and Concentrator-25 columns. Two (2) μg of isolated RNA was reverse transcribed using oligo-dT and MMLV reverse transcriptase. The cDNAs were subjected to PCR on a BioRad CFX96 machine and amplicons were detected using SYBR green. Specific SNX14 primers and HPRT1 primers (reference gene) were used (see Table S1). PCRs were run in a LightCycler 96 Real-Time PCR system (Roche). All the qPCR primers used in this study are listed in Table S1.

### 2.10 FluoZin-3 assay

FluoZin-3 assay was performed 2 days after seeding parental MIN6 cells and MIN6-6 cells (50:1) at <5% confluency. These MIN6-6 cells allow for more accurate determination of focal plane and TIRF angle for the assay. Cells were incubated at 37 °C in low glucose (5.6 mM glucose) KRB (114 mM NaCl, 5 mM KCl, 1.2 mM MgSO4, 1.2 mM KH2PO4, 20 mM HEPES, 2.5 mM CaCl2, 25.5 mM NaHCO3 and 0.2% BSA) for 1.5–2 h with a change of buffer after 1 h. Cell-impermeant FluoZin-3 dye (Thermo Fisher Scientific, Cat#: F24194) in KRB buffer was added to a final concentration of 20 μM. Glucose was added together with the dye to a final concentration of 5.6 mM or 25 mM. Focal plane and TIRF angle were refined after dye addition and the recording (60 ms exposure, no delay) started within 2 min after addition of the dye for 10 min. The videos were processed by substracting each frame from the previous frame. Every five subtracted images were grouped as a max projection through time using the Grouped Z-project function in ImageJ and analyzed at 300 ms time resolution. Individual secretion events or ‘flashes’ were identified using ImageJ software [26] using the point tool. FluoZin-3 movies were blinded then analyzed manually. Cell borders were identified by ROI based on DIC images. Cell number and secretion events within each identified cell were calculated in ImageJ. The detailed protocol has been published previously [27].

Supplementary video related to this article can be found at https://doi.org/10.1016/j.molmet.2022.101541

The following is the supplementary data related to this article:

Multimedia component 1 Multimedia component 1

### 2.11 Immunofluorescence staining

Cultured MIN6 cells on coverslips were treated with experimental conditions for 24 h. The cells were rinsed with ice-cold PBS and fixed with 4% PFA for 10 min at room temperature followed by permeabilization in 0.3% Triton X-100. The cells were then incubated with ITGB1 antiboy (1:200), ITGAV antibody(1:200), vinculin antibody (1:200), paxillin antibody (1:200) and ELKS antibody (1:200) overnight at 4 °C. The cells were subsequently washed with cold PBS three times for 10 min each and incubated with Alexa Fluor 488 or 568–labeled anti-rabbit immunoglobulin G secondary antibody (1:250) (Invitrogen) at room temperature for 2 h. After three washes with cold PBS, cells were stained with Hoechst 33,342 (0.5 μg/mL) and 1.65 μM phalloidin-647 (Biotium). The coverslips were mounted on slides with ProLong Diamond mounting medium (Thermo Fisher Scientific).

### 2.12 Confocal and TIRF imaging

Images were acquired using a Zeiss LSM880 wih Airyscan using a 63 × oil lens. DAPI, Alexa Fluor 488, Alexa Fluor 555, and Alexa Fluor 647 images were acquired sequentially using 405-, 488-, 561-, and 633-nm laser lines. Image quantification was performed using the ImageJ software.

For quantification of the size and number of focal adhesions and ELKS-containing complexes, cells were fixed and stained with ITGB1 antibody and a focal adhesion marker (vinculin or paxillin antibody) or ELKS antibody. Basal plane images were converted to 8-bit and grayscale. Cells were outlined and thresholded to avoid artificial over- or under-filling of areas. Average intensity of ITGB1 per cell, thresholded area per cell and outlined cell area were computed while the number of focal adhesions or ELKS-containing complexes per cell was counted manually. The size of these complexes was calculated by dividing the total thresholded area per cell by the number of the cognate complex per cell [28,29].

Two methods were used to determine the number or density of docked insulin granules. In WT and *Commd3* KD MIN6-6 cells, TIRF detectable phogrin-EGFP marked vesicles were defined as docked insulin granules. The cells were imaged using a Nikon TIRF2 System for TE2000 with a TIRF 100 × 1.49 NA oil immersion lens. The granules were counted with the ImageJ software.

To determine the number of stably docked vesicles, pCMV-NPY-EGFP transfected WT and *Commd3* KD MIN6 cells were preincubated with 5.6 mM glucose for 1h. After replacing the medium with freshly made KRB buffer containing 5.6 mM, the cells were imaged on a TIRF microscope for 5 min at 1 frame/s. The first and last 5 frames were averaged. The averaged images were compared to identify insulin granules that remained in the same vicinity. These granules were considered as stably docked.

To determine the release probability of insulin granules, pCMV-IAPP-Emerald transfected WT and *Commd3* KD MIN6 cells were preincubated in medium with 5.6 mM glucose for 1h. After replacing the medium with freshly made KRB buffer containing 5.6 mM, the cells were imaged on a Nikon TIRF2 System for TE2000 with a TIRF 100 × 1.49 NA oil immersion lens for 10 min at 1 frame/s. A secretion event was defined as a sudden disappearance (with in 1 s) of a docked vesicle.

### 2.13 Statistics

Results are expressed as the mean ± SEM unless otherwise specified. For data sets where the distribution was appropriate (normal distribution as determined by the D'Agostino & Pearson omnibus normality test), statistics were calculated by one-way ANOVA with Tukey's multiple comparisons test. When the data distribution was non-normal, a Kruskal–Wallis nonparametric and multiple comparison test was used (>2 data sets). Means were compared using Student's t test when two experimental conditions were compared. GraphPad Prism 9 was used for statistical analyses and graphical representations. Significance was defined at p ≤ 0.05.

## 3 Results

### 3.1 Development of a MIN6 reporter cell line for bulk and single cell GSIS assay

A high throughput single cell assay of exocytosis of dense core granules (insulin granules) is essential for a genomewide CRISPR/Cas9 screen for genes that regulate GSIS. We chose to use a lumenal SNAP tag in combination with membrane impermeant fluorescently labeled SNAP substrates to label exocytic Insulin granules. SNAP is a 20 kDa engineered form of the DNA repair protein O6-alkylguanine-DNA alkyltransferase that reacts specifically and rapidly with benzylguanine (BG) derivatives to form an irreversible covalent conjugation [30]. As the secretory granules fuse with the plasma membrane to secrete insulin, they also expose their lumen to the extracellular environment. To anchor the SNAP tag in the lumen of Insulin granules, we inserted it into the lumenal region immediately downstream of the dibasic proteolytic site of the granule-specific integral membrane protein, phogrin [31]. Although its function in insulin secretion is still not clearly defined, phogrin has been used to anchor various sensors to the lumen of secretory granules [[32], [33], [34]]. To easily identify cells that express SNAP-Phogrin, we also fused enhanced green fluorescent protein (EGFP) to the cytoplasmic tail of phogrin. This allows selection and visualization of cells that express SNAP-Phogrin-EGFP fusion protein.

**Figure 1:**
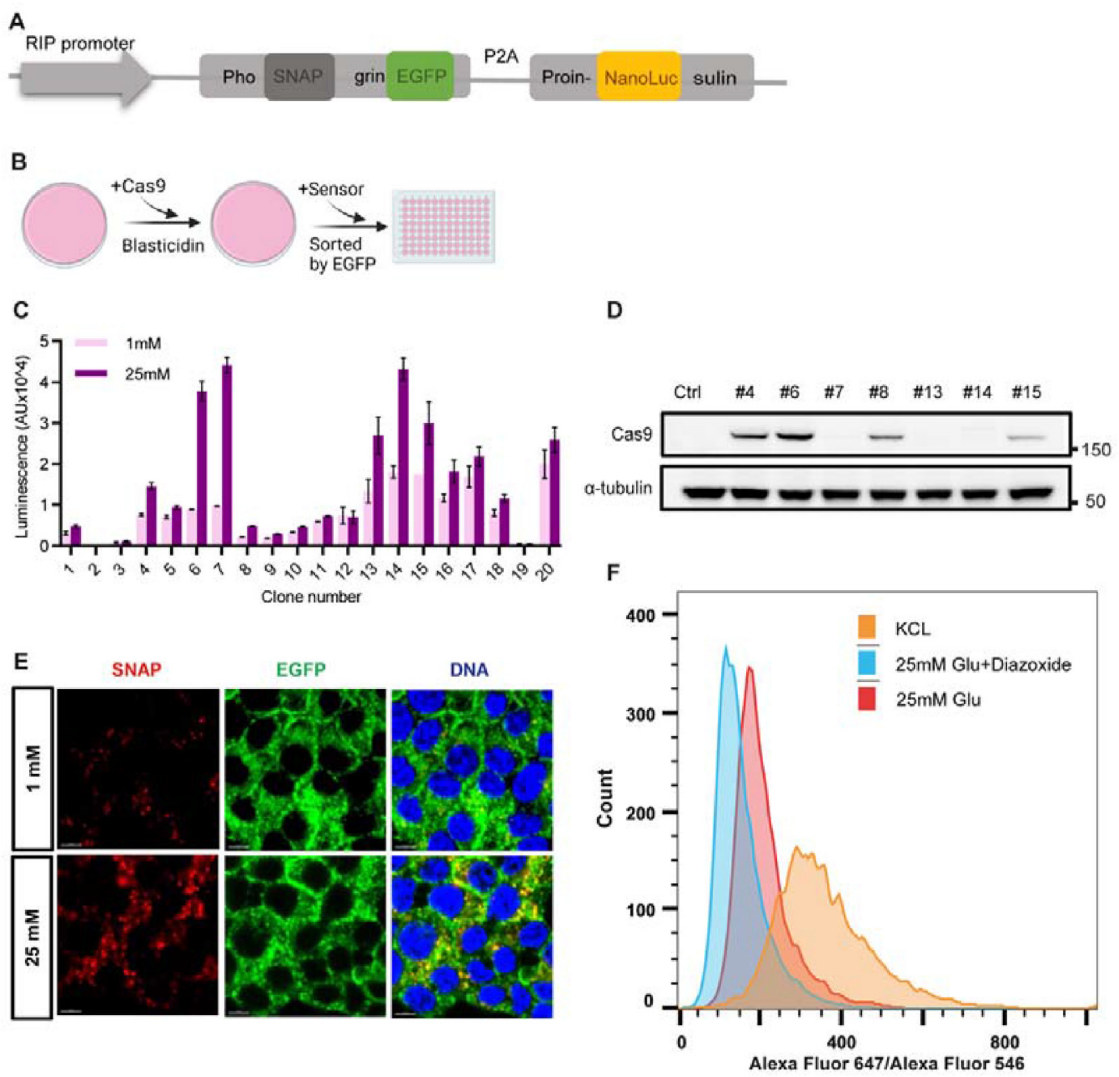
**Development of a MIN6 reporter cell line.** (A) Schematic of the reporter construct (Created with BioRender.com). (B) The workflow of establishing reporter cell clones. (C) Characterization of glucose induced insulin secretion in 20 clones. The luminescence intensity of NanoLuc was detected after 30min stimulation of 1 mM glucose (black, n = 4) or 25 mM glucose (gray, n = 4). (D) Immunoblot of Cas9 protein in control cells and clone #4, #6, #7, #8, #13, #14 and #15. (E) Representative confocal images of MIN6-6 after 20 min incubation with SNAP surface dye Alexa Fluor 546 together with 1 mM glucose or 25 mM glucose. Phogrin-EGFP was also imaged. Scale bar, 5 μm. (F) Flow cytometry analysis of MIN6-6 cells labeled with Alexa Fluor 546 in 1 mM glucose for 20 min, followed by labeling with Alexa Fluor 647 in 25 mM glucose (red), 25 mM glucose plus 300 μM Diazoxide (blue), or 40 mM KCl (orange).

Many beta cell clones need to be tested to identify one with appropriate glucose responsiveness. This is because most beta cell lines consist of cells with heterogenous glucose responsiveness [35,36]. To facilitate clone selection, we also linked the coding sequence for a modified preproinsulin (unprocessed insulin) to that of SNAP-Phogrin-EGFP with a viral 2A sequence. The viral 2A peptide causes ribosome skipping, allowing production of multiple independent proteins from one transcript [37]. The preproinsulin gene was modified by replacing the coding sequence for the non-functional C chain with that of nano-luciferase (NanoLuc) [38,39], the smallest luciferase with the strongest bioluminescence. NanoLuc will be packaged together and co-secreted with insulin. Thus, nanoLuc activity in the medium, which can be easily measured with high sensitivity, is a surrogate for secreted insulin. Therefore, NanoLuc allows assaying of GSIS activity in a large number of samples [38]. The reporter gene is under the control of the rat insulin promoter (Rip) as depicted in Figure 1A and was inserted in a lentiviral vector.

We chose MIN6 cells as the target for our CRISPR screen. MIN6 cells are derived from mouse insulinoma [40]. Early passage (<40) MIN6 cells are glucose responsive and have simple culture conditions [40,41]. Early passage MIN6 cells were transduced with viral particles of the construct and a Cas9-expressing construct, Lenti-Cas9-2A-Blast [42] (Figure1B). After two weeks of selection, EGFP-positive cells were FACS sorted into two 96 well plates to generate clones. NanoLuc assays were performed to characterize glucose induction index of each subclone (Figure 1C, Fig. S1A). Seven subclones with a glucose induction index >2 fold were expanded and tested for the expression levels of Cas9 by western blot. The #6 subclone was chosen for additional experiments because it showed both a high GSIS index (Figure 1C) and high Cas9 expression (Figure 1D). We named this clone MIN6-6. When MIN6-6 cells were incubated sequentially in 1 mM glucose and 25 mM glucose for 30 min each, the nanoLuc activity in the latter was more than 4 fold higher, or a GSIS index >4. With a 1h incubation, the GSIS index was 10, similar to the GSIS index of 11 determined by an insulin ELISA assay (Figs. S1B–C). These results indicate that MIN6-6 is glucose responsive and the nanoLuc secretion assay is a viable surrogate for insulin secretion.

We next determined whether SNAP labeling is also glucose responsive. After queching potential surface SNAP with a non-fluorescent cell impermeant SNAP substrate, MIN6-6 cells were exposed to medium with 1 mM or 25 mM glucose containing a cell-impermeant SNAP surface dye Alexa Fluor 546. After 20 min, cells were fixed and imaged with a confocal microscope. The signal of the SNAP label was much higher in MIN6-6 cells in high glucose medium than these in low glucose medium (Figure 1E). In addition, the SNAP labels were mostly intracellular and colocalized with EGFP positive granules, probably due to compensentary endocytosis. Parental MIN6 cells without the reporter showed little SNAP labeling either in low or high glucose medium (Figs. S1C–D), indicating labeling specificity.

Last, we determined whether MIN6-6 cells are compatible with the high throughput GSIS assay in single cells. After quenching, MIN6-6 cells were sequentially incubated in low glucose medium with SNAP surface dye Alexa Fluor 546 then in experimental media with SNAP surface dye Alexa Fluor 647 for 20 min each before being fixed. The experimental media contained either 25 mM glucose, 25 mM glucose plus 300 μM diazoxide, or 40 mM KCl. Diazoxide is an KATP channel opener that inhibits ATP-induced membrane depolarization while KCl induces maximal insulin secretion in the absence of glucose. The fixed cells were analyzed by flow cytometry. The mean fluorescence intensity (MFI) ratio of Alexa Fluor 647/546 was used to measure glucose response index. Compared to cells stimulated with 25 mM Glucose, KCl right shifted the MFI distribution while diazoxide left shifted the MFI distribution (Figure1F). These data confirmed that SNAP labeling intensity positively correlates with insulin secretion. MIN6-6 cells are therefore suitable for a genomewide CRISPR screen for genes affecting insulin secretion.

### 3.2 A genome-wide CRISPR-Cas9 screen identifies a role for the commander complex in insulin secretion

**Figure 2:**
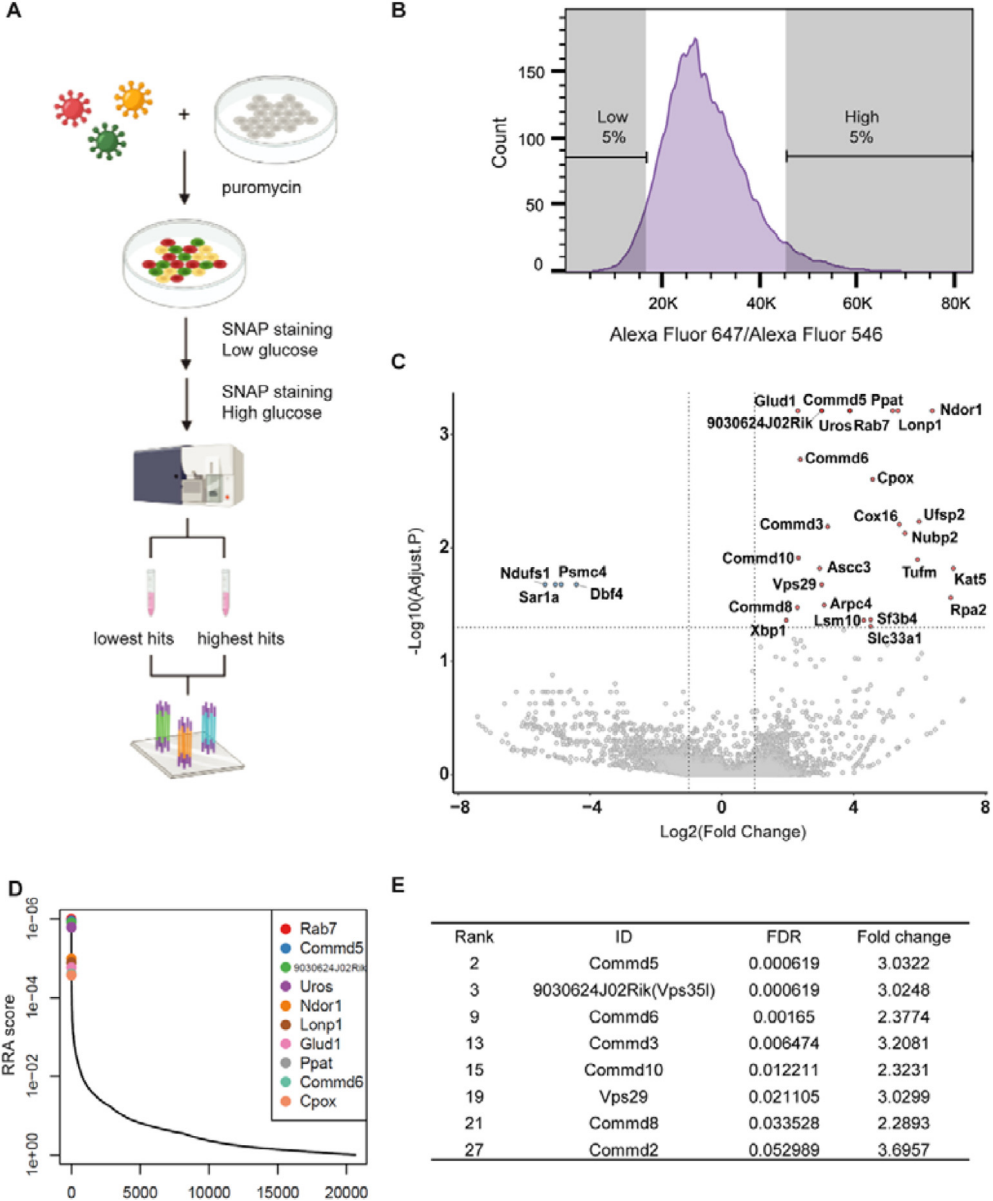
**A genome-wide screen identifies regulators of GSIS.** (A) The workflow of the screen. A genome-wide CRISPR sgRNA lentiviral library (78,637 gRNAs) was used to infect Cas9-expressing MIN6-6 cells at low MOI (0.3–0.4). After selection, most cells should express only one sgRNA. CRISPR/Cas9-edited cells were sequentially labeled with Alexa Fluor 546 in 1 mM glucose and Alexa Fluor 647 in 25 mM glucose. They were then subjected to FACS. Sorted cells were lysed for genomic DNA extraction, sgRNA amplification and next generation sequencing. (B) Top and bottom 5% cells were sorted as high and low glucose induction index according to their Alexa Fluor 546/Alexa Fluor 647 ratio. (C) A volcano plot demonstrating the FDR and fold change-based ranking of genes identified in the cells with high glucose induction index. (D) Candidate gene ranking by robust rank aggregation (RRA) scores calculated by MAGeCK. A smaller RRA score indicates more essentiality. Commander complex members and genes known to regulation insulin secretion are highlighted. (E) Table listing the Commander complex members discovered in the CRISPR screen with their rank, FDR and fold change. FDR, False discovery rate.

To screen for genes important for insulin secretion, we first established a MIN6-6 mutant library using the Mouse CRISPR Knockout Pooled Library (Brie), containing 78,637 gRNAs targeting 19,674 genes and 1,000 non-targeting control sgRNAs [25]. After a two-week puromycin selection, the cells were harvested. Some cells (∼8x10^7^) were used for genomic DNA to determine the sgRNA distribution, while some were used in two independent screens to identify regulators of insulin secretion. For the screen, the cells were cultured in 1 mM glucose DMEM for 4h, followed by a 20 min incubation with SNAP-Surface Block. They were then sequentially labeled with SNAP Surface Alexa Fluor 546 in 1 mM glucose DMEM for 20 min, followed by SNAP Surface Alexa Fluor 647 in 25 mM glucose DMEM for another 20 min. Cells were dissociated and fixed for fluorescence-activated cell sorting (FACS). Cells displaying the highest or lowest 5% Alexa Fluor 647/546 MFI ratio were collected (Figure 2A–B). The genomic DNA from these cells was isolated and the sgRNA representation was quantified by deep sequencing. Quality control showed >88% sgRNAs were present. Gini index showed an even distribution in the control sgRNA library. In the selected pools, higher cell numbers resulted in a more even distribution (Figs. S2A–C). The abundance of many sgRNAs were substantially increased in both replicates compared with unsorted control population. Nontargeting control sgRNAs exhibited no enrichment (Figure 2C), confirming that the screens were effective and specific. Genes were ranked based on the enrichment of their targeting sgRNAs using the MAGeCK (model-based analysis of genome-wide CRISPR-Cas9 knockout) algorithm [43]. The sgRNA of a few dozens of genes were enriched in the pool with increased glucose stimulation index. Some were known negative GSIS regulators such as Glud1 and Rab7. However, only a few genes whose sgRNAs were enriched in the pool with decreased glucose stimulation index (Fig. S2D). The small number of hits may be due to the highly stringent cell selection.

We focused on gene hits in the pool of high glucose stimulation index because these may be drug targets. Several of these genes encode enzymes catalyzing lipid or carbohydrate metabolism, mediators of gene expression, proteins important for vesicular trafficking, as well as genes lacking annotated functions. Several members of Commander complex were among the top gene hits. These include COMM Domain Containing 5 (COMMD5), VPS35 Endosomal Protein Sorting Factor Like (VPS35L), COMM Domain Containing 6 (COMMD6), COMM Domain Containing 3 (COMMD3), COMM Domain Containing 10 (COMMD10), VPS29 Retromer Complex Component (VPS29), COMM Domain Containing 8 (COMMD8), and COMM Domain Containing 2 (COMMD2) (Figure 2D–E). The identification of multiple components of the Commander complex provides strong support for its role in insulin secretion.

### 3.3 Loss of *Commd3* decreases basal insulin secretion

The Commander complex is important for endosomal recycling of a subset of proteins. It comprises 15 different proteins, including COMMD 1–10, CCDC93, CCDC22, VPS35L, VPS26C, and VPS29. COMMD 1–10 are thought to always exist in a complex, and loss of one lowers the expression of the rest [44]. Mutations in *Commd* genes are linked to human RS/3C syndrome, a developmental disease in humans [45]. *Commd4* and *Commd7* have been implicated in T2D in two genome-wide association studies [46,47]. However, the role of Commander complex in pancreatic beta cells has not been characterized.

**Figure 3:**
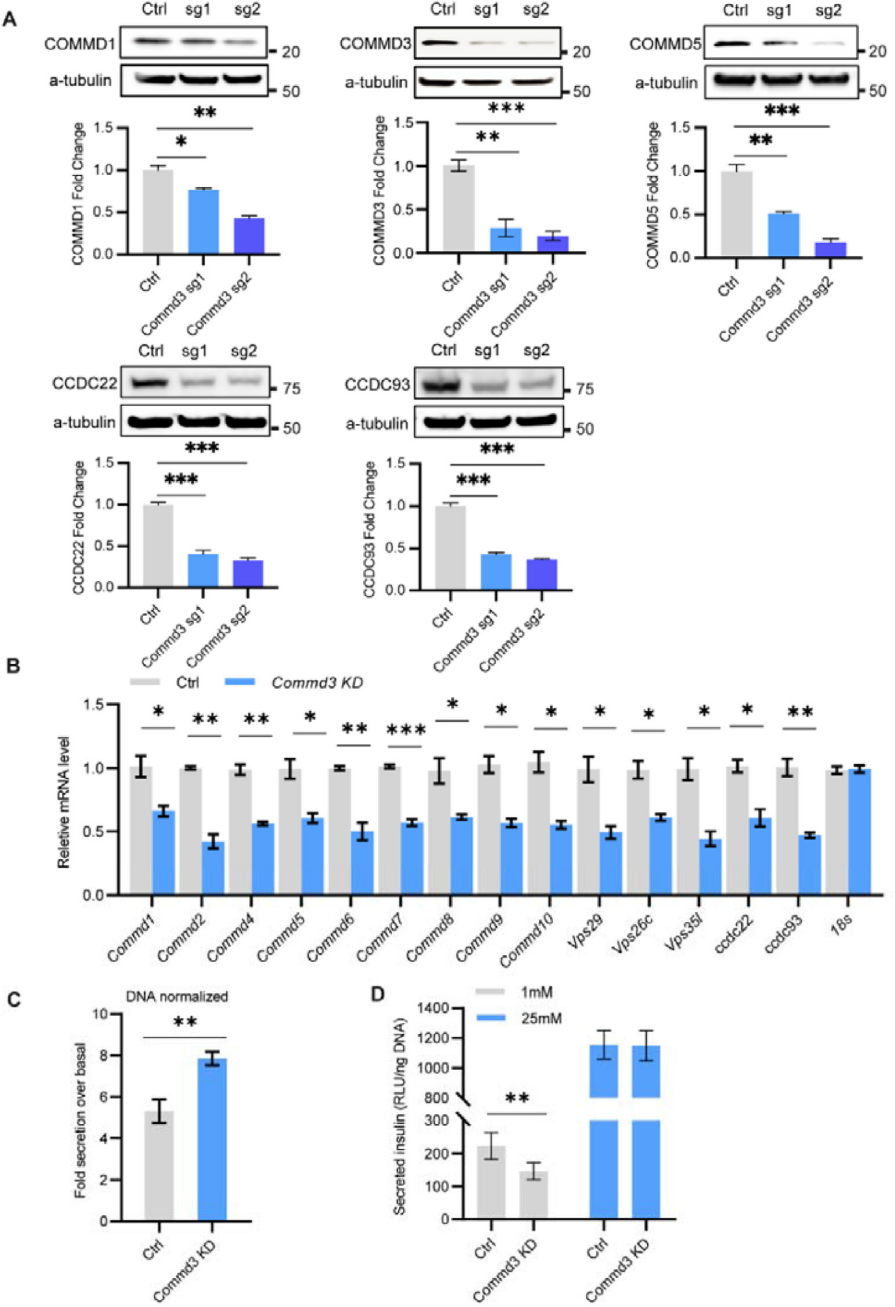
**The Commander complex enhances basal insulin secretion.** (A) Validation of *Commd3* KD cells. Two sgRNA targeting *Commd3* genes were designed to deplete COMMD3 using CRISPR/Cas9. Western blot analysis of COMMD1, COMMD3, COMMD5, CCDC22, CCDC93 and α-Tubulin in control cells and *Commd3* KD cells (control, sg1, sg2). Quantification of band intensity of each protein relative to α-tubulin. Error bar, S.E.M. (B) Quantitative RT- PCR analysis of mRNA levels of Commander complex family members in control and *Commd3* KD cells from two independent experiments. (C) Glucose induction index of control and *Commd3* KD MIN6-6 cells. (D) Basal (1 mM glucose) and stimulated (25 mM glucose) insulin secretion (using nanoLuc as a surrogate) in control and *Commd3* KD cells. Mann–Whitney nonparametric comparison ∗∗∗P < 0.001, ∗∗P < 0.01, ∗P < 0.05.

We first validated that loss of the Commander complex in beta cells improves glcuose stimulation index. We generated two *Commd3* KD populations in MIN6 and MIN6-6 cells using CRISPR/Cas9 with two different sgRNAs. Western blot analysis indicated that COMMD3 protein levels were decreased in both populations, with sgRNA2 population having 80–90% reduction (Figure 3B). In addition, 4 other Commander complex components, COMMD1, COMMD5, CCDC22, and CCDC93 were also reduced by 60–85% in the sgRNA2 population (Figure 3A), consistent with previous studies [48,49]. These cells are referred to as *Commd3* KD cells. Interestingly, qRT-PCR analysis of genes for the Commander complex in control and *Commd3* KD cells revealed a 40–60% reduction in the *Commd3* KD cells compared to control MIN6 cells (Figure 3B). The results suggest a feedback control at the level of either transcription or mRNA stability. *Commd3* KD cells showed a high glucose stimulation index, validating the screen (Figure 3C). Moreover, this increase of GSIS index is mainly due to a decreased basal insulin secretion but not stimulated insulin secretion (Figure 3D). The glucose response curve showed less insulin secretion at 1 mM and 5.6 mM glucose but a normal insulin secretion at 11.1 mM and 25 mM glucose, indicating COMMD3 regulates basal insulin secretion (Fig. S3A). We confirmed that this is not specific to *Commd3* KD, we also generated *Commd5* KD cells. Similar to *Commd3* KD cells, *Commd5* KD cells also have a lower glucose induction index due to lower basal insulin secretion (Figs. S3C–E). To determine whether the low basal secretion is due to increased beta cell maturity, we compared the transcript levels of multiple maturation markers by qRT-PCR. No change was detected in the *Commd3* KD cells (Fig. S3B). Therefore, the Commander complex enhances basal insulin secretion without affecting beta cell maturity.

### 3.4 Loss of *Commd3* reduces the number of exocytic events and docked insulin granules

**Figure 4:**
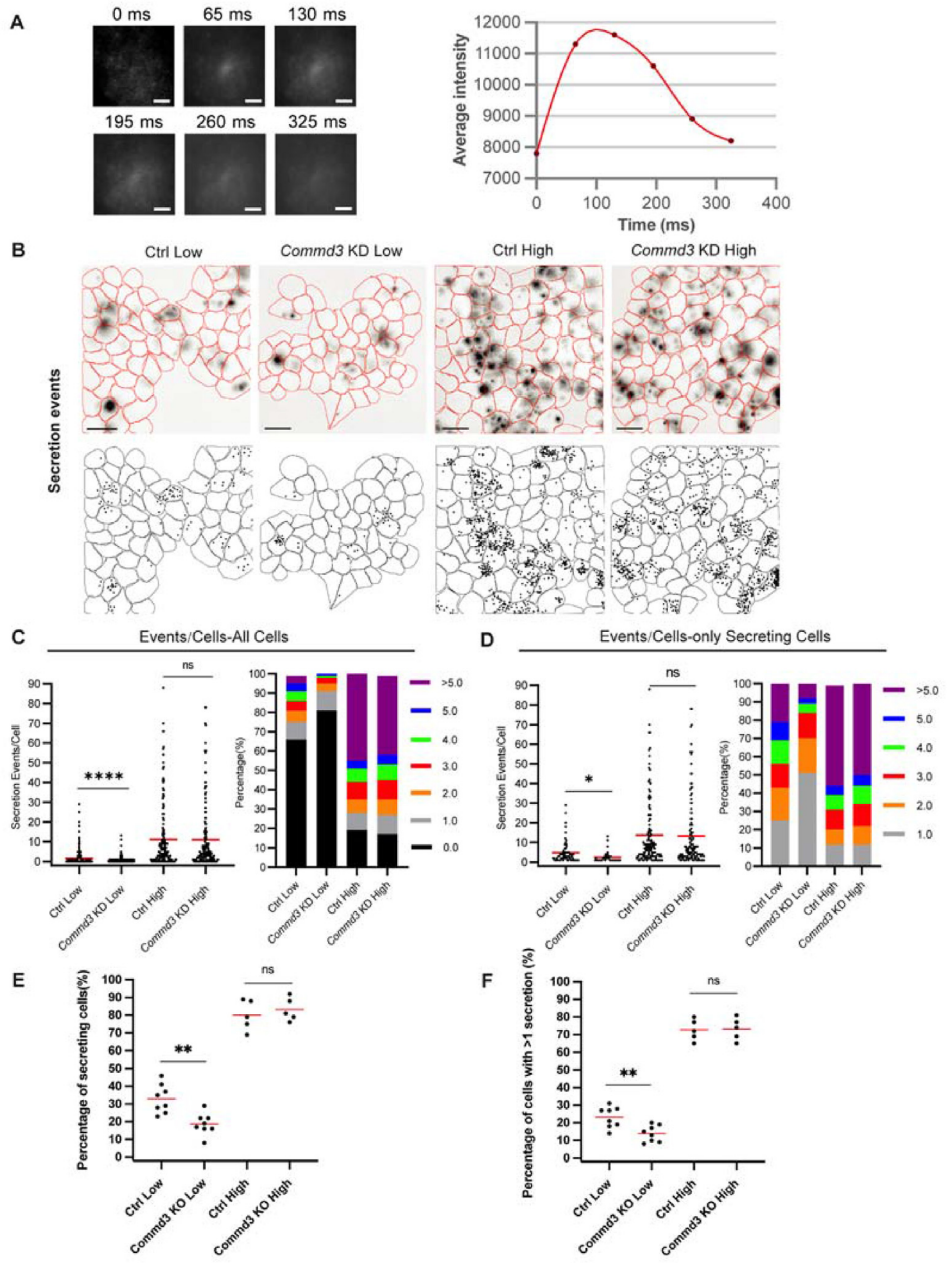
**Loss of *Commd3* decreases the insulin secretion events at basal glucose level.** (A) Representative images and average intensity of a single secretion event prior to image processing. Scale bars: 3 μm. (B) Inverted intensity time projections of all secretion events during the duration of the FluoZin-3 assay. Upper panels. Accumulation of FluoZin-3 flashes are represented as black areas. Cell border (in red) was identified via DIC images (see Materials and methods). Lower panel. Schematic rendering of the upper panel showing cell outlines (black lines) and secretion events (black dots). Control and *Commd3* KD cells were preincubated in 5.6 mM glucose medium for 1h and followed by incubation with 5.6 mM or 25 mM glucose. Scale bars: 60 μm. (C) Left panel, graph showing secretion events per cell at basal condition as detected by the FluoZin-3 assay. All cells in a field of view are analyzed, regardless of their activity during the assay. Red bar, mean. Kruskal–Wallis nonparametric and multiple comparison test. N = 158–200 cells from 5 to 8 dishes. Right panel, stacked histogram of the percentage of cells that had different number of secretion events. (D) Left panel, graph of secretion events in stimulated cells during the assay. Red bar, mean. Kruskal–Wallis nonparametric and multiple comparison test. N = 68–131 cells from 5 to 8 dishes. Right panel, stacked histogram of the percentage of cells that had different number of secretion events. (E) Graph of the percentage of cells with at least 1 secretion event in a field of view. Red bars, mean. One-way ANOVA and multiple comparison tests. (F) Graph of the percentage of cells with more than one secretion event in a field of view. Red bars, mean. One-way ANOVA and multiple comparison tests. ∗∗∗∗P < 0.0001, ∗∗P < 0.01, ∗P < 0.05.

To understand the underlying mechanism of decreased basal insulin secretion, we used FluoZin-3 based live imaging on a total internal reflection fluorescence (TIRF) microscope [27,50] to examine insulin granule docking and secretion in control and *Commd 3* KD MIN6 cells. FluoZin-3 increases fluorescence emission upon binding to Zn^2+^, a component of insulin crystals in the insulin granules. And due to the cell impermeability of FluoZin-3, it only binds Zn^2+^ after secretin. A “flush” of fluorescence occurs with each exocytic event (Figure 4A). FluoZin-3 was added into the KRB buffer with 5.6 mM or 25 mM glucose and the cells were imaged for 10 min at a rate of 60 ms/frame. At 5.6 mM glucose, we detected insulin secretion in a lower fraction of *Commd3* KD cells (20%) than in control MIN6 cells (30%) (Figure 4B–E). In addition, there were fewer secretion events on average in active *Commd3* KD cells than in control cells (Figure 4B–D,F). At 25 mM glucose, there was no difference in either the fraction of active cells or average secretion events per cell between *Commd 3* KD and control cells (Figure 4B–D). These results support that loss of *Commd3* decreases basal but not glucose-stimulated insulin secretion.

**Figure 5:**
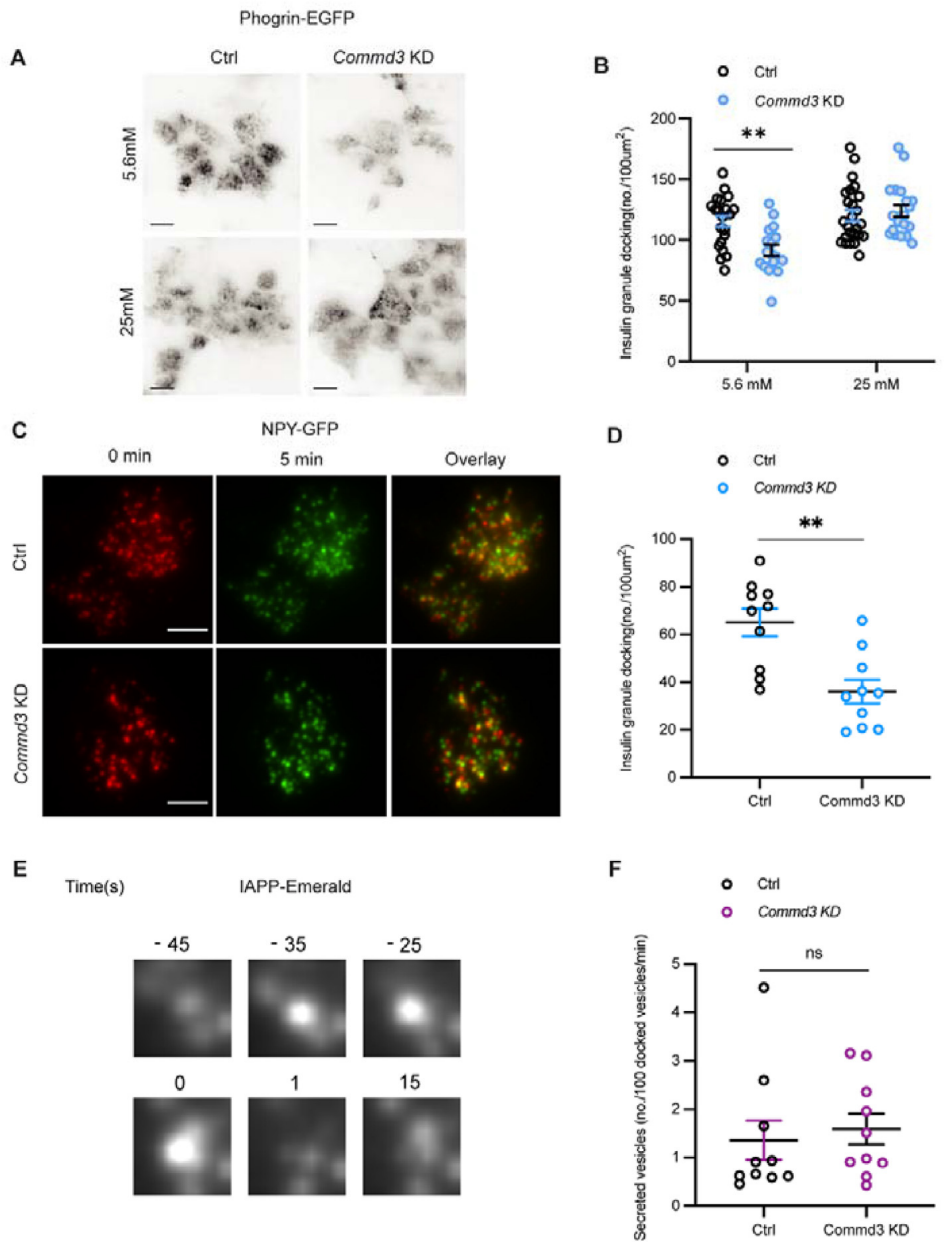
**Loss of Commd3 reduces docked insulin vesicles at basal glucose levels.** (A) Representative TIRF images showing cortical insulin granules based on phogrin-EGFP signal. Control and *Commd3* KD MIN6-6 cells were preincubated in medium with 5.6 mM glucose for 1h followed by medium with 5.6 mM or 25 mM glucose for 1h and fixed immediately. Scale bar, 20 μm. (B) Quantification of docked insulin vesicles in control and *Commd3* KD MIN6-6 cells treated as in (A). Kruskal–Wallis nonparametric and multiple comparison test. Single data points are plotted. Horizontal line, mean; error bars, S.E.M. (C) Representative TIRF images taken 5 min apart for identification of stably docked insulin granules. NPY-EGFP transfected WT and *Commd3* KD MIN6 cells were preincubated with KRB buffer containing 5.6 mM glucose for 1h. The medium was then replaced with fresh medium. The cells were imaged on a TIRF microscope for 5 min at 1 frame/s. The first (red) and last (green) 5 frames were averaged. The two images were overlayed to identify insulin granules that remained in the same vicinity. These granules were considered as stably docked. Scale bar, 5 μm. (D) Quantification of stably docked insulin vesicles in control and *Commd3* KD MIN6 cells treated as in (C). Kruskal–Wallis nonparametric test. Single data points are plotted. Horizontal line, mean. Error bars, S.E.M. (E) Representative images showing the exocytosis of an insulin granule. IAPP-Emerald transfected WT and *Commd3* KD MIN6 cells were preincubated with KRB buffer containing 5.6 mM glucose for 1h and medium was refreshed. The cells were imaged on a TIRF microscope for 10 min at 1 frame/s. (F) Quantification of secretion events normalized to the number of stably docked granules in control and *Commd3* KD MIN6 cells treated as in (E). Kruskal–Wallis nonparametric test. Single data points are plotted. Horizontal line, mean. Error bars, S.E.M. ns, no significant difference. ∗∗P < 0.01. TIRFM, total internal reflection fluorescence microscope.

A decrease in basal insulin secretion could result from a decreased fusion probability or a reduced number of docked granules [51]. To investigate if the decreased basal secretion is due to fewer docked insulin granules in *Commd3* KD cells, we measured the number of vesicles near the basal membrane in the SNAP-phogrin-EGFP expressing MIN6-6 cells using TIRF microscopy. We found a significant reduction of the density of cortical insulin granules in *Commd3* KD MIN6-6 cells compared to the control MIN6-6 cells at 5.6 mM glucose. After 1h of glucose stimulation, however, no difference was detected in the density of cortical insulin granules between *Commd3* KD and control MIN6-6 cells (Figure 5A–B). As vesicle docking is dynamic [52], some of the cortical insulin granules may be transiently docked, we therefore compared the number of insulin granules that remain docked for 5 min in pCMV-NPY-EGFP transfected control and *Commd3* KD MIN6 cells using TIRF microscopy. There was a significant decrease of stably docked insulin granules in *Commd3* KD cells compared to control cells (Figure 5C–D).

To determine whether a decreased release probability also contributes to the lower basal secretion, we determined the number of exocytic granules in pCMV-IAPP-Emerald transfected control and *Commd3* KD MIN6 cells. Exocytosis is indicated by a sudden increase followed by a disappearance of fluorescence (with in 1 s) [53,54]. There was no significant difference between *Commd3* KD and control MIN6 cells (Figure 5E–F, Video 1). Together, these results suggest the decreased basal insulin secretion in *Commd3* KD cells is due to fewer docked insulin granules.

### 3.5 Loss of *Commd3* causes retention of ITGB1 in Rab4-positive recycling endosomes

**Figure 6:**
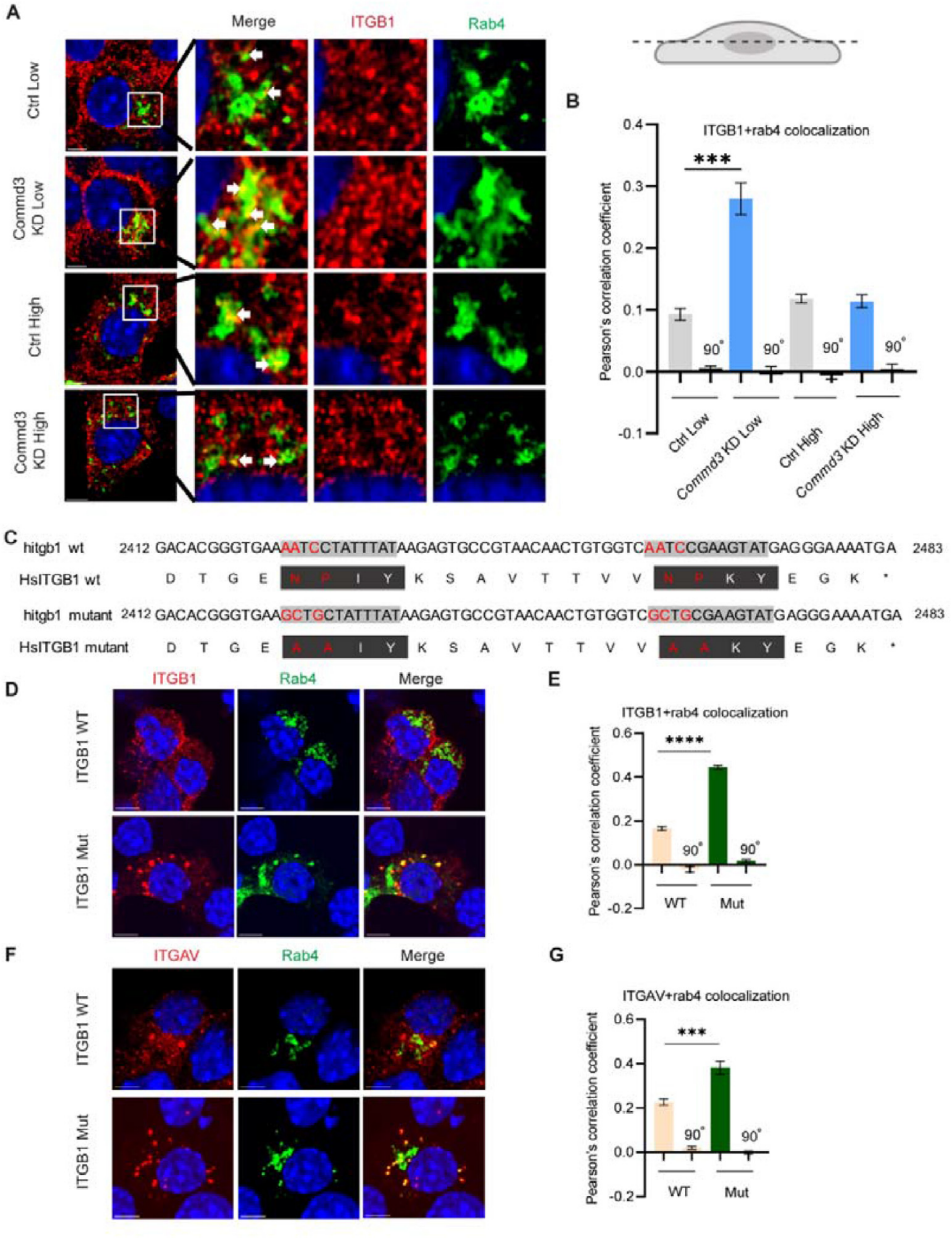
**Loss of *Commd3* causes retention of ITGB1 in Rab4-positive recycling endosomes.** (A) Representative images of ITGB1 and Rab4 subcellular localization. Control and *Commd3* KD MIN6 cells were transfected with Rab-4-EGFP, cultured in medium with 5.6 mM or 25 mM glucose, then fixed for ITGB1 immunofluorescence (red) and DNA stain (blue). The cells were imaged with a confocal microscope. White arrow, the colocalization of ITGB1 and Rab4. Scale bar, 3 μm. (B) Quantification of colocalization of ITGB1 and Rab4 in control and *Commd3* KD MIN6 cells using Pearson's correlation coefficient between the two channels. N = 18–19 images from three independent experiments; 90° indicates a 90° rotation of one image channel to rule out nonspecific colocalization. Error bars, S.E.M. (C) Schematic showing WT and mutant ITGB1 sequences. Two NPxY motifs were mutated to AAxY. (D) Representative images of Rab4 and ITGB1 subcellular localization. Cells were treated as in (A) except that they were also transfected with WT or mutant ITGB1-expressing construct. Scale bar, 3 μm. (E) Quantification of ITGB1-Rab4 colocalization in MIN6 cells transfected with ITGB1 WT or ITGB1 mut using Pearson's correlation coefficient between the two channels. N = 15–20 images from three independent experiments. Error bars, S.E.M. (F) Representative images of ITGAV (red) and Rab4 (green) subcellular localization in MIN6 cells transfected with WT or mutant ITGB1-expressing construct. Cells were treated as in (A). Scale bar, 3 μm. (G) Quantification of ITGAV-Rab4 colocalization in MIN6 cells transfected with WT or mutant ITGB1-expressing construct using Pearson's correlation coefficient between the two channels. N = 15–18 images from three independent experiments. Error bars, S.E.M.

Having demonstrated that *Commd3* KD decreases basal insulin secretion due to fewer docked insulin granules, we next investigated the underlying molecular mechanism. Commander complex associates with SNX17, which mediates recycling of membrane proteins with a ΦxNPxY or ΦxNxxY sorting motif, such as ITGB1, LDLR and LRP1 [55]. *Ldlr* is expressed in MIN6 cells and is important for cholesterol uptake [56]. Cholesterol is important for the formation of Syntaxin clusters on the plasma membrane, where secretory vesicles preferentially dock [57]. Defective LDLR recycling therefore could impair insulin granules docking. However, β cell function is normal in *Ldlr*−/− mice [56]. Therefore, we excluded defective LDLR recycling as the mechanism for the decreased basal secretion in the *Commd3* deficient cells. Singla et al. reported that knocking down *Commd3*, *Ccdc93*, *Vps35l* or *Vps26c* causes ITGB1 accumulation in WASH-positive intracellular vesicles in HeLa cells [48]. Mice with ITGB1 ablation in beta cells have decreased beta cell mass and impaired GSIS [16,58,59]. The role of ITGB1 recycling in insulin secretion has not been reported. Therefore, we investigated whether *Commd3* KD affects ITGB1 endocytic trafficking in MIN6 cells by using EGFP-tagged Rab4, 5, 7, and 11 to mark different endocytic compartments. At 5.6 mM glucose, there was extensive colocalization of ITGB1 and Rab4 in *Commd3* KD cells, in contrast to the low colocalization in control MIN6 cells (Figure 6A–B), indicating that ITGB1 proteins are stuck in the Rab4-positive early endosome or recycling endosomes. These results are consistent with a recycling defect in ITGB1 trafficking. At 25 mM glucose, however, the colocalization of ITGB1 and Rab4 was low in both *Commd3* KD cells and control MIN6 cells, indicating the activation of a different recycling mechanism. There was no difference in ITGB1 colocalization with Rab5, Rab7 or Rab11 between *Commd3* KD cells and control cells in both basal glucose and high glucose conditions (Fig. S4).

### 3.6 Defective ITGB1 recycling impairs insulin granule docking and basal insulin secretion

We next investigated whether the decreased basal insulin secretion and insulin granule docking in *Commd3* KD cells is due to defective SNX17-mediated ITGB1 recycling. We mutated the ΦxNPxY motifs of human ITGB1 (Figure 6C) and performed cotransfection to co-express Rab4-EGFP with either the wild type or the mutant ITGB1 in MIN6 cells. We found that, just as in the *Commd3* KD cells, there was an increase of ITGB1 in Rab4-positive vesicles in cells expressing the mutant ITGB1 compared to those expressing the wild type ITGB1 (Figure 6D–E). As ITGB1 can dimerize with at least 12 α subunits [60], we examined whether endogenous α subunits are also retained in the Rab4-positive vesicles. Integrin AV transcript is the most abundant α integrin in MIN6 cells [61]. In MIN6 cells expressing mutant ITGB1, ITGAV was also sequestered in the Rab4-positive vesicles as expected (Figure 6F–G).

**Figure 7:**
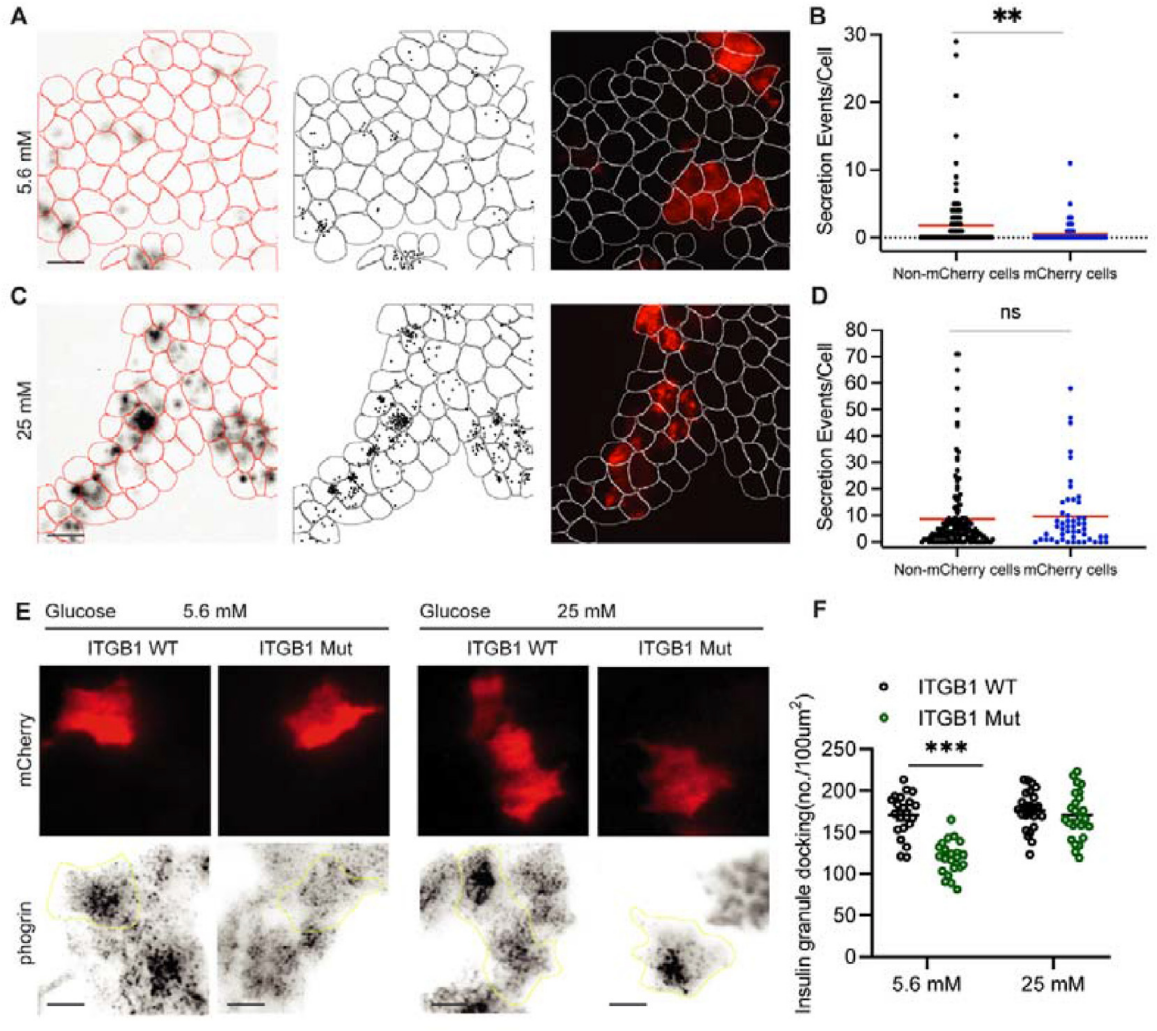
**Defective ITGB1 recycling impairs insulin granule docking and basal insulin secretion.** (A) Insulin secretion events detected by FluoZin-3 in MIN6 cells co-expressing mCherry and mutant ITGB1 and their untransfected neighbors. After transfection, the cells were preincubated in 5.6 mM glucose medium for 1h followed by fresh medium with 5.6 mM mCherry indicates transfected cells. Scale bars, 60 μm. (B) Quantification of secretion events per cell detected by FluoZin-3 assay at 5.6 mM glucose. All cells in a field of view were analyzed regardless of transfection status. Red bar, mean. Kruskal–Wallis nonparametric and multiple comparison test. N = 75 mCherry -cells and 200 non-mCherry cells from 5 to 10 dishes. (C) Representative images showing insulin secretion detected by FluoZin-3 in MIN6 cells expressing co-expressing mCherry and mutant ITGB1. MIN6 cells expressing exogenous WT or mutant ITGB1 were preincubated in 5.6 mM glucose medium for 1h followed by fresh medium with 25 mM glucose. mCherry-cells indicated transfected cells. Scale bars: 60 μm. (D) Quantification of secretion events per cell detected by FluoZin-3 assay at 25 mM glucose. N = 50 mCherry-cells and 150 non-mCherry cells from 5 to 10 dishes. (E) Docked insulin granules in MIN6-6 cells co-expressing mCherry and WT or mutant ITGB1. Top panel. Images showing transfected (red) and untransfected cells. Bottom panel. TIRF images showing insulin granules (Phogrin-EGFP positive vesicles). After transfection (48h), cells were preincubated in 5.6 mM glucose medium for 1h, stimulated with 25 mM glucose medium for 1h and fixed immediately. mCherry-cells indicated transfected cells. Scale bar, 10 μm. (F) Quantification of docked insulin vesicles in MIN6-6 cells expressing WT or mutant ITGB1 analyzed as in (E). N = 21–25 cells. Kruskal–Wallis nonparametric and multiple comparison test. Single data points are plotted. Horizontal line, mean. Error bars, S.E.M. ns, no significant difference, ∗∗∗P < 0.0001, ∗∗P < 0.01.

To determine whether the mutant ITGB1 affects insulin secretion, we co-transfected expression vectors for mCherry and mutant ITGB1 in MIN6 cells. We then performed the FluoZin-3 assay to quantify the insulin secretion events under 5.6 mM and 25 mM glucose in transfected and untransfected cells. At 5.6 mM glucose, we found a much lower percentage of cells that secrete insulin compared to untransfected cells. The average secretion events in cells expressing the mutant ITGB1 were also decreased compared to untransfected cells (Figure 7A–B). However, at 25 mM glucose, the percentage of secreting cells and secretion events per cell were similar between transfected and untransfected cells (Figure 7C–D).

We further investigated whether expression of the mutant ITGB1 reduces insulin granule docking. We measured the number of docked vesicles near the basal membrane in MIN6 cells co-expressing wild type or mutant ITGB1 with mCherry. At 5.6 mM glucose, the insulin granule density near the basal membrane was decreased in MIN6 cells expressing the mutant ITGB1 compared to these expressing the wildtype ITGB1. After 1h of glucose stimulation, however, no difference was detected in the number of insulin granules between cells expressing the mutant and wild type ITGB1 (Figure 7E–F). Taken together, these results indicate that *Commd3* KD cells have defective ITGB1 recycling at basal glucose conditions, which decreases insulin secretion and insulin granule docking.

### 3.7 Defective ITGB1 recycling reduces cortical focal adhesions

**Figure 8:**
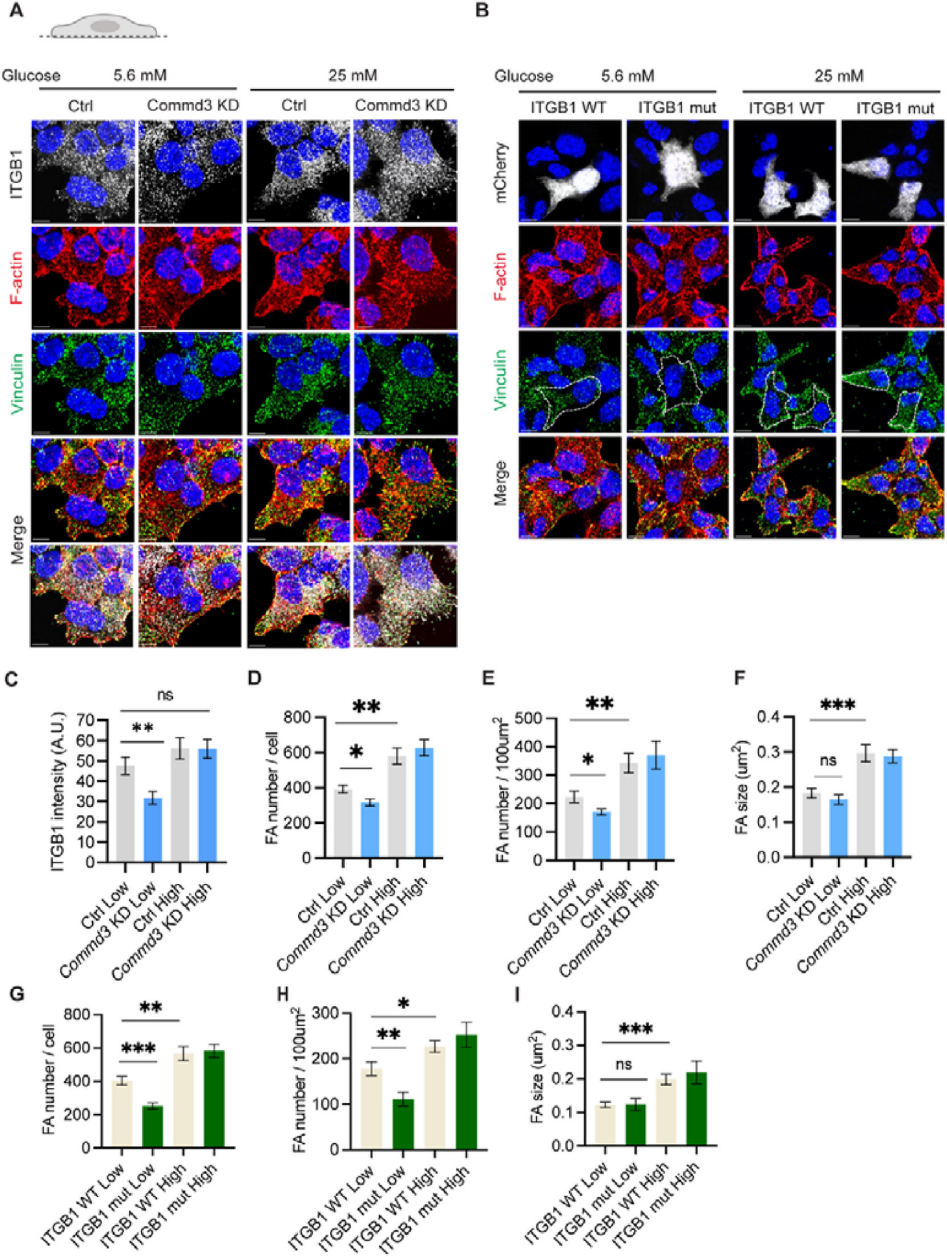
**Defective ITGB1 recycling reduces cortical focal adhesions.** (A) Representative images of ITGB1, F-actin, and Vinculin localization at basal plasma membrane of control and *Commd3* KD MIN6 cells. The cells were cultured in medium containing 5.6 mM or 25 mM glucose, fixed, stained for ITGB1 (white), F-actin (red) and Vinculin (green) and imaged with confocal microscopy. (B) Representative images of F-actin and Vinculin localization at basal plasma membrane of MIN6 cells co-expressing mCherry and WT or mutant ITGB1. The cells were transfected, cultured in medium containing 5.6 mM or 25 mM glucose, fixed, stained for F-actin (red) and Vinculin (green) and imaged with confocal microscopy. White cells indicate transfected cells. (C) Quantification of fluorescence intensity of the ITGB1 signal in control and *Commd3* KD MIN6 cells treated as in (A). (D) Quantification of focal adhesion number per cell in control and *Commd3* KD MIN6 cells treated as in (A). (E) Quantification of focal adhesion number per 100 μm^2^ area in control and *Commd3* KD MIN6 cells treated as in (A). (F) Quantification of focal adhesion size in control and *Commd3* KD MIN6 cells treated as in (A). (G) Quantification of focal adhesion number per cell in MIN6 cells expressing WT or mutant ITGB1 treated as in (B). (H) Quantification of focal adhesion number per 100 μm^2^ area in MIN6 cells expressing WT or mutant ITGB1 treated as in (B). (I) Quantification of focal adhesion size in MIN6 cells expressing WT or mutant ITGB1 treated as in (B). ns, no significant difference; ∗∗∗P < 0.001, ∗∗P < 0.01, ∗P < 0.05, unpaired t test. Error bars, S.E.M.

How defective ITGB1 recycling reduces insulin granule docking and insulin secretion is unknown. Integrin recycling regulates focal adhesion remodeling [62], which is important for GSIS [63]. Therefore, we investigated if the defective ITGB1 recycling from *Commd3* deficiency affects focal adhesions. In *Commd3* KD cells, the ITGB1 in the basal PM was significantly lower than control cells as shown by immunofluorescence (Figure 8A,C; Fig. 5). Moreover, the number of focal adhesions was significantly decreased at basal glucose level in *Commd3* KD cells compared to control cells. However, 20 min after stimulation with 25 mM glucose, the number of focal adhesions in *Commd3* KD cells became indistinguishable from control cells (Figure 8A,D-E; Figure 5A, C-D). There was no difference in the size of focal adhesions between *Commd3* KD and control cells under either basal or high glucose condition (Figure 8A,F; Figs. S5A and E). Similarly, MIN6 cells overexpressing the mutant ITGB1 had fewer focal adhesions than cells expressing the wildtype ITGB1 at basal status, but not after 20 min of 25 mM glucose stimulation (Figure 8B,G-I; Fig. S5B, F–H). These results suggest a role of diminished cortical focal adhesions in the reduced insulin granule docking and basal insulin secretion.

### 3.8 Reduced cortical focal adhesions diminish the assembly of ELKS complexes

**Figure 9:**
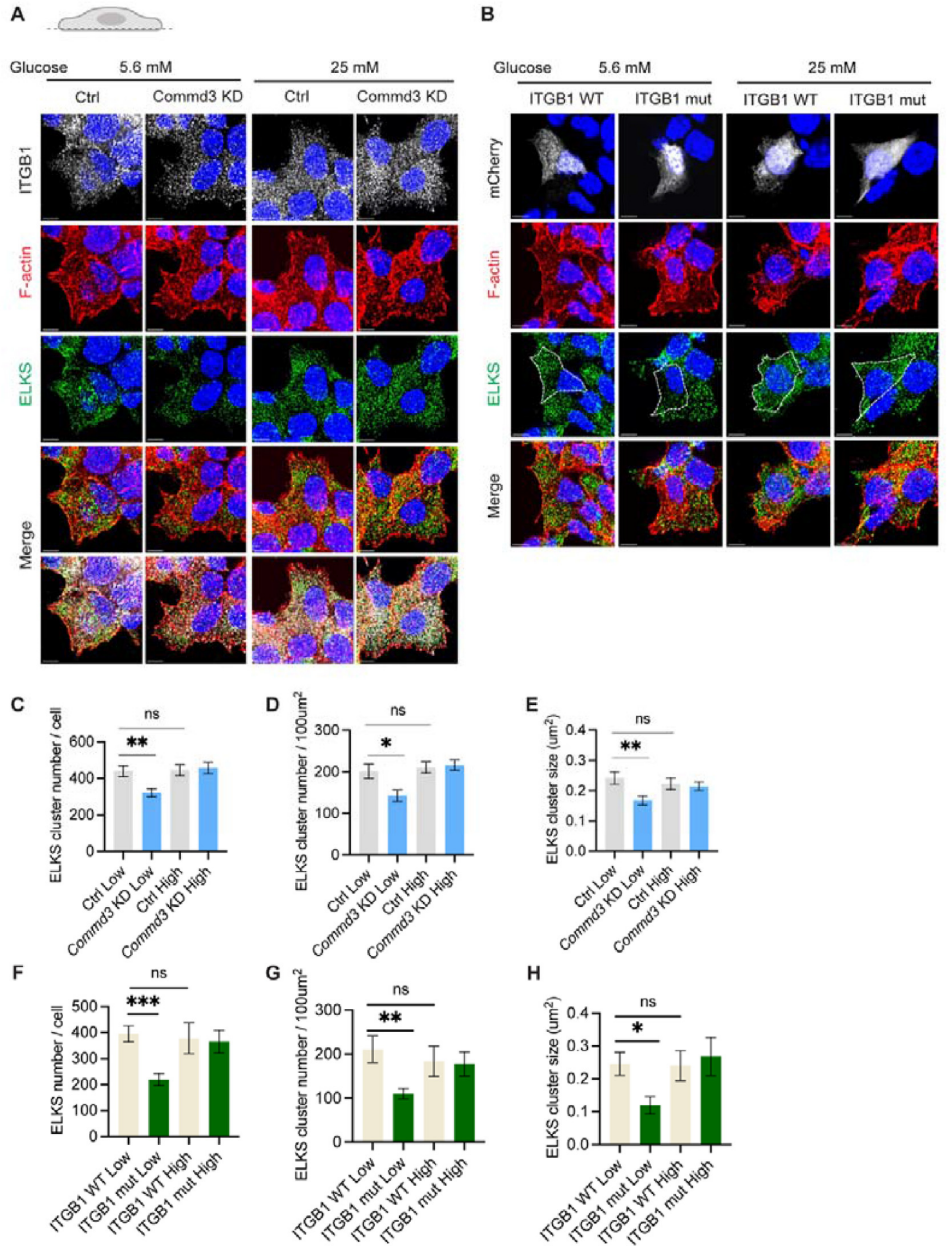
**Reduced cortical focal adhesions diminishes the assembly of ELKS complexes.** (A) Representative images of ITGB1, F-actin, and ELKS localization at basal plasma membrane of control and *Commd3* KD MIN6 cells. The cells were cultured in medium containing 5.6 mM or 25 mM glucose, fixed, staining for ITGB1 (white), F-actin (red) and ELKS (green) and imaged with confocal microscopy. (B) Representative images of F-actin and ELKS localization at basal plasma membrane of MIN6 cells co-expressing mCherry and WT or mutant ITGB1. The cells were transfected, cultured in medium containing 5.6 mM or 25 mM glucose, fixed, stained for F-actin (red) and ELKS (green) and imaged with confocal microscopy. (C) Quantification of ELKS cluster number per cell in control and *Commd3* KD MIN6 cells treated as in (A). (D) Quantification of ELKS cluster number per 100 μm^2^ area in control and *Commd3* KD MIN6 cells treated as in (A). (E) Quantification of ELKS cluster size in control and *Commd3* KD MIN6 cells treated as in (A). (F) Quantification of ELKS cluster number per cell in MIN6 cells expressing WT or mutant ITGB1 treated as in (B). (G) Quantification of ELKS cluster number per 100 μm^2^ area in MIN6 cells transfected with ITGB1 WT or ITGB1 mut treated as in (B). (H) Quantification of ELKS cluster size in MIN6 cells expressing WT or mutant ITGB1 treated as in (B). ns, no significant difference; ∗∗∗P < 0.001, ∗∗P < 0.01, ∗P < 0.05, unpaired t test. Error bars, S.E.M.

Focal adhesions plays an important role in polarized insulin secretion [18]. The underlying mechanism remains unclear but may relate to the ITGB1 mediated assembly of a complex consisting of Bassoon, Piccolo, RIMs, ELKS and liprins that are important for vesicle docking and exocytosis [64,65]. ITGB1 has been shown to recruit ELKS, an important scaffold of the complex, through its interaction with LL5β and Talin-KANK1 [66,67]. ELKS is important for insulin granule docking and secretion in beta cells [21,23]. Therefore, we compared cortical ELKS in *Commd3* KD and control cells. The number of ELKS clusters was decreased and their size smaller at basal glucose compared to control cells. After 20 min high glucose stimulation, however, the number and size of ELKS clusters were indistinguishable in *Commd3* KD and control cells (Figure 9A,C-E). Similarly, in cells expressing the recycling-defective ITGB1, there were fewer and smaller ELKS clusters than in untransfected cells or cells expressing the wildtype ITGB1 at basal glucose condition. However, the difference disappeared after 20 min of high glucose stimulation (Figure 9B,F–H). These data suggest that decreased ELKS-containing complexes may be responsible for fewer docked insulin granules and decreased basal insulin secretion.

Taken together, our results demonstrated a previously unknown function of the Commander complex in basal insulin secretion. We showed that by recycling ITGB1, Commander complex increases cortical adhesions, which enhances the assembly of the ELKS-containing complexes. The resulting increase of the number of insulin granules near the palsma membrane strengthens basal insulin secretion.

## 4 Discussion

In the present work, we established a highly glucose responsive beta cell reporter line (MIN6-6) with multiple engineered functionalities. This line allows for CRISPR/Cas9 mutagenesis, quantification of bulk insulin secretion by a straightforward NanoLuc assay, and en masse quantification of glucose-stimulated insulin granule exocytosis in single cells. We conducted a genome-wide screen for genes affecting glucose stimulation index. Several components of the Commander complex were among the top hits of cells with increased glucose stimulation index. We demonstrated that loss of function of the Commander complex decreases basal glucose secretion with little effect on GSIS. The decreased basal insulin secretion was due, at least in part, to fewer docked insulin granules. The reduction in docked granules partially results from impaired ITGB1 recycling and the consequent reduction of cortical focal adhesions and ELKS-containing complexes.

The screen identified several genes known to regulate insulin secretion, demonstrating the validity of the assay. For example, an activating mutation in Glud1 causes congenital hyperinsulinism [68]. Beta cells with increased Glud1 activity have increased basal but blunted glucose-stimulated insulin secretion [69,70], consistent with an increased glucose stimulation index in cells with lower Glud1 activity. Similarly, shRNA-mediated Rab7 knockdown increases glucose-stimulated insulin secretion [71], consistent with the screen results. Interestingly, a number of genes encoding non-respiratory components of the mitochondria were also among the top hits in the pool of increased glucose induction index. Whether they affect basal or stimulated insulin secretion remains to be determined. Only limited genes were identified in the pool of decreased glucose induction index, however. This may be a result of the heterogeneity of the glucose responsiveness of the cells despite their clonal origin, the small shift in the distribution of the glucose induction index (Figure 1F), and the stringent selection we implemented (Figure 2B). A less stringent selection may allow identification of more genes.

The screen identified multiple components of the Commander complex, which consists of 15 subunits. COMMD1-10, along with CCDC93, can all interact with CCDC22 to form the CCC complex. The CCC complex interacts with the retriever complex, consisting of Vps35l, Vps26c and Vps29, to form the Commander complex [45]. Although some of the COMMD proteins may have preferred binding partners [72], they can all bind to each other promiscuously [44]. All 15 genes are expressed in MIN6 cells. Interestingly, knockdown of *Commd3* led to not only a reduction of protein levels of other components, but also downregulation of mRNA levels of all other genes in the complex, suggesting feedback regulation at transcription or mRNA stability (Figure 3A–B). Previously, it has been reported that hepatocyte-specific knockout of Commd1, Commd6, or Commd9 to a lesser extent, destabilizes all Commander components [49]. Similar results have been seen in HeLa cells [48]. Our results indicate that the downregulation may be at least partially due to a decrease in mRNA levels.

We found that the increased glucose stimulation index in *Commd3* mutant cells results from lower basal insulin secretion. Both NanoLuc assay of bulk insulin secretion and FluoZin-3 assay of insulin secretion in single cells indicated a lower basal secretion and normal glucose-stimulated secretion in *Commd3* mutant cells (Figure 3D, Figure 4B–D). Although lower basal secretion is often a sign of maturity [73], there was no difference in the expression of several maturity markers (Fig. S3B). Instead, we found fewer docked insulin granules at basal state in the *Commd3* mutant cells (Figure 5A–D). This contrasts with granuphilin mutant beta cells where a decrease of docked granules slightly enhances basal insulin secretion [74]. The difference is likely due to the dual roles of granuphilin in docking and exocytosis [74,75]. COMMD3 probably does not play a direct role in either docking or exocytosis. The normal glucose-stimulated insulin secretion in *Commd3* mutant cells indicates it does not affect glucose-stimulated granule docking nor exocytosis [76].

COMMD3 has been reported to regulate ITGB1 recycling and deficiency of COMMD3 causes ITGB1 accumulation in WASH complex in HeLa cells [48]. We found in C*ommd3* mutant MIN6 cells, ITGB1 accumulated in Rab4 vesicles at basal glucose (Figure 6A). This was recapitulated by expressing a recycling-deficient ITGB1 in *Commd3*-intact MIN6 cells. Expression of the recycling-deficient ITGB1 also reduced insulin secretion and insulin granule docking at basal condition but not at high glucose stimulation. ITGB1 can be recycled via different pathways depending on its partner, activation state, and cellular physiology [77]. Our results suggest that Commander-mediated recycling from Rab4-positive endosomes is the predominant pathway in beta cells at basal glucose conditions and that high glucose activates other ITGB1 recycling pathways.

The role of ITGB1 in basal insulin secretion is not clear. In two studies from the Wang lab on mice with beta cell specific loss of *Itgb1,* it was initially shown that the male mutant mice have normal fasting glucose levels but glucose intolerance, while female mutants are normal in fasting glucose and glucose tolerance [17]. In a follow up study, however, it was shown that primary islets from males, but not female, have decreased basal insulin secretion [58], similar to our results in MIN6 cells. The discrepancy may be due to the more complex regulation of insulin secretion in vivo.

Integrin mediates cell adhesion through membrane associated focal adhesions that connect to actin and the microtubule cytoskeleton [78]. As expected, impairment of ITGB1 recycling decreases membrane ITGB1 clusters and focal adhesions at basal glucose conditions (Figs. 8 and S5). Several studies have found a role for focal adhesions in GSIS [63,79,80]. Yet, how reduced focal adhesions leads to a reduction of basal insulin secretion is unknown. Genetic or pharmacological inhibition of focal adhesion kinase (FAK) reduces insulin granule docking without affecting basal insulin secretion [80,81]. It is possible that the focal adhesion effect on basal insulin secretion is FAK independent. How a reduced number of focal adhesions decreases insulin granules docking is also unknown. In the case of FAK deficiency, the diminishment of insulin granule docking is thought to be a result of impaired actin depolymerization [80,81]. In *Commd3* deficient MIN6 cells, our results suggest a decreased number and size of cortical ELKS-containing complexes as a potential mechanism. In high glucose condition, ELKS promotes exocytosis by anchoring insulin granules to the vicinity of VDCC [23]. It does not appear to occur at basal glucose conditions as no change of granule release probability was observed in *Commd3* KD cells (Figure 5E–F). Other described mechanisms of ELKS action may operate at basal glucose. The ELKS-containing complexes may promote the delivery of insulin granules by anchoring CMSCs to the focal adhesions [67], target the granules to the exocytic complexes through LL5β-Kank1 interaction with focal adhesions [5], and/or facilitate the docking of insulin granules [22]. Although the role of ELKS in GSIS has been well established [65], its role in basal insulin secretion needs further investigations.

In summary, in a screen to identify genes that affect the glucose induction index of insulin secretion in beta cells, we identify a role for the Commander complex in enhancing basal insulin secretion. Mechanistically, we show that the Commander complex promotes docking of insulin granules at basal state. This is mediated, at least in part, by its function in ITGB1 recycling. Defects in ITGB1 recycling reduce its membrane distribution and the number of focal adhesions. Detailed molecular mechanisms connecting focal adhesions to insulin granule docking and secretion at basal glucose conditions remain to be elucidated.

## Author's contrbution

W.Chen conceived the project. L. Yang carried out most experiments. M.A.Fye and K. Bracy assisted FluoZin-3 assay and TIRF imaging. B. Yang and Z. Tang participated in confocal imaging and analysis. Y. Zhang assisted in bioinformatic analysis. L. Yang and W. Chen wrote and edited the manuscript. S. Haigh and B.A.Covington edited the manuscript and provided advice. I. Kevarina provided materials and advice, S. Qu and W. Chen oversaw the project.

## Figures and Tables

**Suppl Fig s1:**
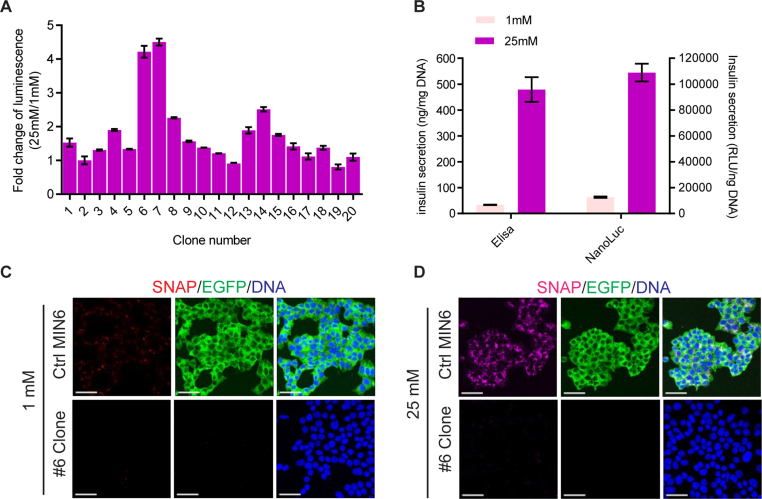
**Development of a MIN6 reporter cell line.** (A) Characterization of glucose stimulated insulin secretion in 20 clones. The luminescent intensity of NanoLuc was detected after 30 min stimulation with 1 mM glucose (black, n=4) or 25 mM glucose (gray, n=4). The fold change was the ratio of luminescence detected in high glucose medium over that in low glucose medium. (B) Comparison of the insulin secretion measured by an ELISA kit and the NanoGlo kit. The same samples were measured with both insulin Elisa kit and NanoGlo Luciferase kit. (C) Confocal image of parental MIN6 cells and MIN6-6 cells after 20 min incubation with SNAP surface dye Alexa Fluor 546 together with 1 mM glucose or SNAP surface dye Alexa Fluor 647 with 25 mM glucose (D) Phogrin-EGFP was also imaged. Scale bar, 25 µm.

**Suppl Fig s2:**
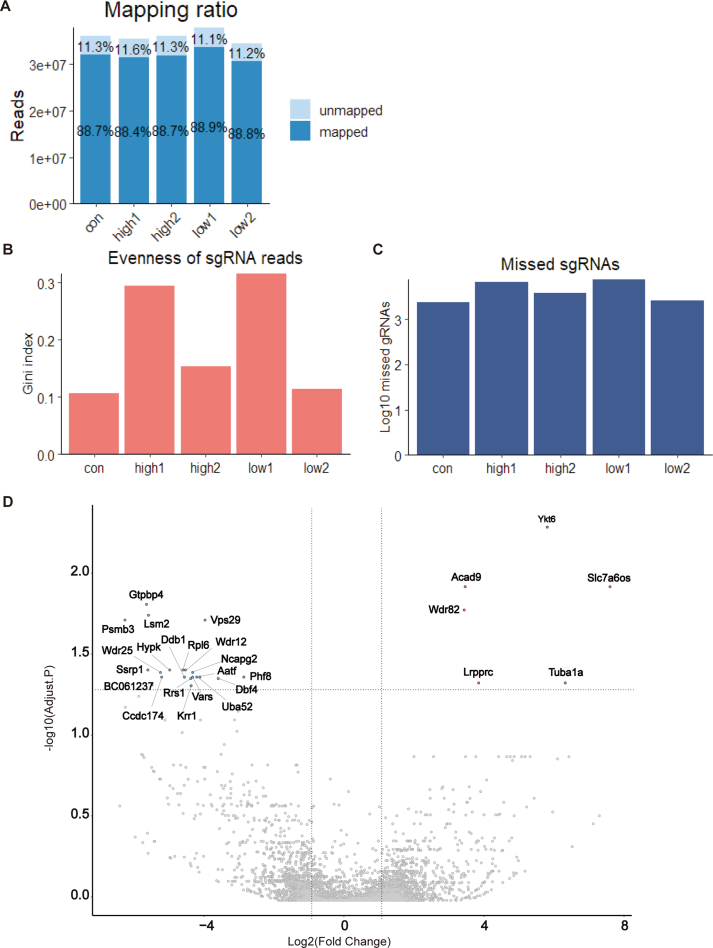
**Genome-wide CRISPR-Cas9 screen using MIN6-6 cells to identify regulators for insulin secretion.** (A) Read counts and mapping percentages. The mapped read percentage is greater than 88%. (B) The Gini index of each sample, which measures the evenness of read depth within samples. (C) Number of missed sgRNAs in each sample. (D) Volcano plot demonstrating the ranking of genes in the low GSIS cells based on the FDR and fold change.

**Suppl Fig s3:**
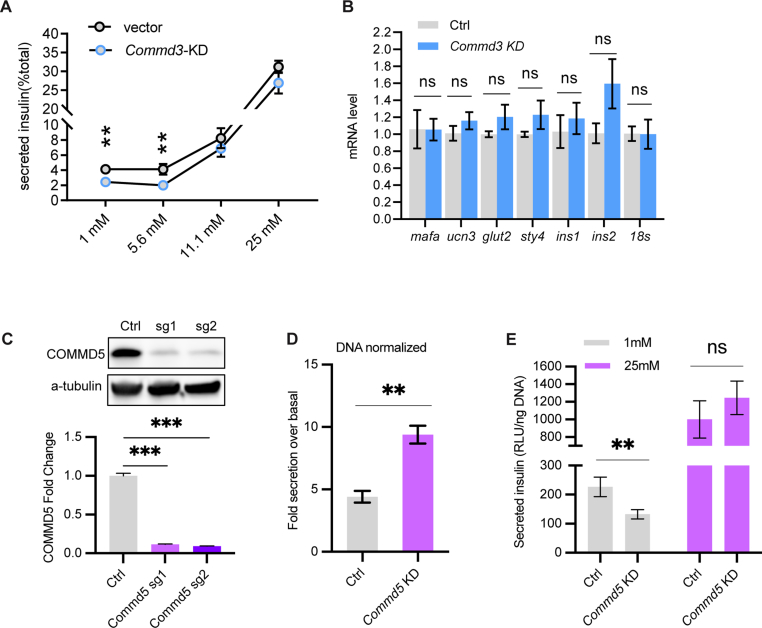
**Characterization of *Commd3* KD MIN6 cells.** (A) Glucose response curve of control and *Commd3* KD MIN6 cells. (B) Quantitative RT-PCR analysis of maturation markers in control and *Commd3* KD MIN6 cells. (C) Western blot analysis showing COMMD5 levels in 3 MIN6 cell populations transduced with CRISPR virus expressing no sgRNA or one of 2 sgRNAs targeting *Commd5* genes. The cells were designated as control, sg1, and sg2, respectively. α-Tubulin was used as loading control. Quantification of band intensities of COMMD5 versus α-tubulin. Error bar, S.E.M. (D) Glucose induction index of control and *Commd5* KD MIN6-6 cells. (E) Basal (1 mM glucose) and stimulated (25 mM glucose) insulin secretion in control and *Commd5* KD MIN6-6 cells. Mann-Whitney nonparametric comparison, ∗∗∗P < 0.001, ∗∗P < 0.01.

**Suppl Fig s4:**
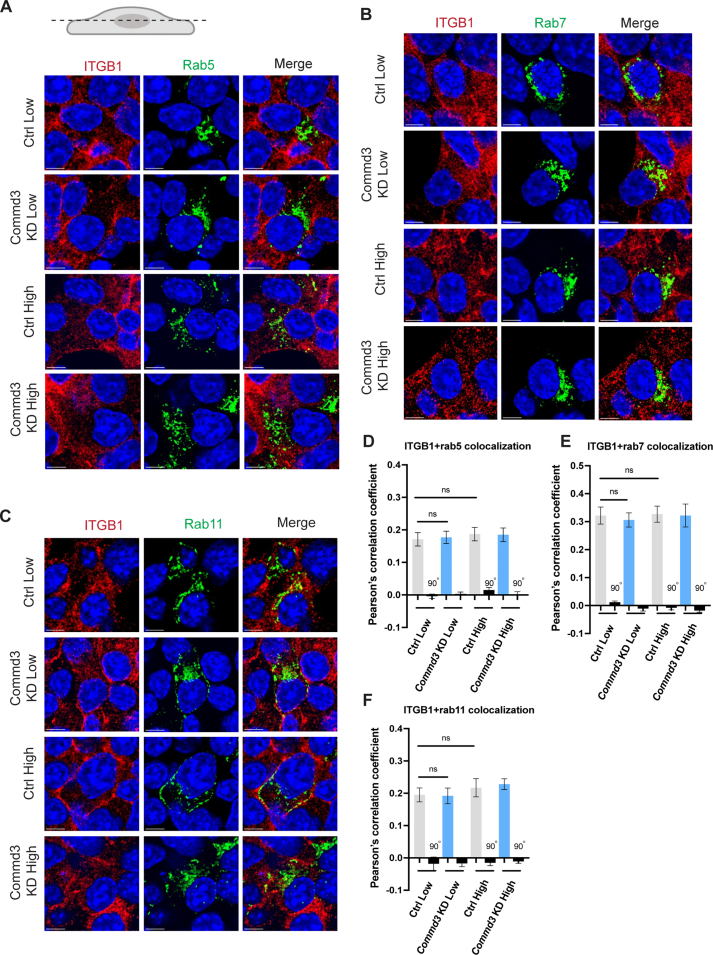
**Loss of *Commd3* does not cause retention of ITGB1 in Rab5, Rab7 or Rab11-positive endosomes.** (A) Representative images of ITGB1 and Rab5 localization at midplane of control and *Commd3* KD MIN6 cells. The cells were transfected with a Rab5-expressing construct, cultured in medium containing 5.6 mM or 25 mM glucose, fixed, staining for ITGB1 (red) and DNA (blue) and imaged with confocal microscopy. Scale bar, 3 µm (B) Representative images of ITGB1 and Rab7 localization at midplane of control and *Commd3* KD MIN6 cells. The cells were transfected with a Rab7-expressing construct, cultured in medium containing 5.6 mM or 25 mM glucose, fixed, staining for ITGB1 (red) and DNA (blue) and imaged with confocal microscopy. Scale bar, 3 µm. (C) Representative images of ITGB1 and Rab11 localization at midplane of control and *Commd3* KD MIN6 cells. The cells were transfected with a Rab11-expressing construct, cultured in medium containing 5.6 mM or 25 mM glucose, fixed, staining for ITGB1 (red) and DNA (blue) and imaged with confocal microscopy. Scale bar, 3 µm. (D) Quantification of colocalization between ITGB1 and Rab5 in control and *Commd3* KD MIN6 cells using Pearson’s correlation coefficient between the two channels. N=10 images from three independent experiments. (E) Quantification of colocalization between ITGB1 and Rab7 in control and *Commd3* KD MIN6 cells using Pearson’s correlation coefficient between the two channels. N=10 images from three independent experiments. (F) Quantification of colocalization between ITGB1 and Rab11 in control and *Commd3* KD MIN6 cells using Pearson’s correlation coefficient between the two channels. N=10 images from three independent experiments; 90° indicates a 90° rotation of one image channel to rule out nonspecific colocalization. Error bars, S.E.M.

**Suppl Fig s5:**
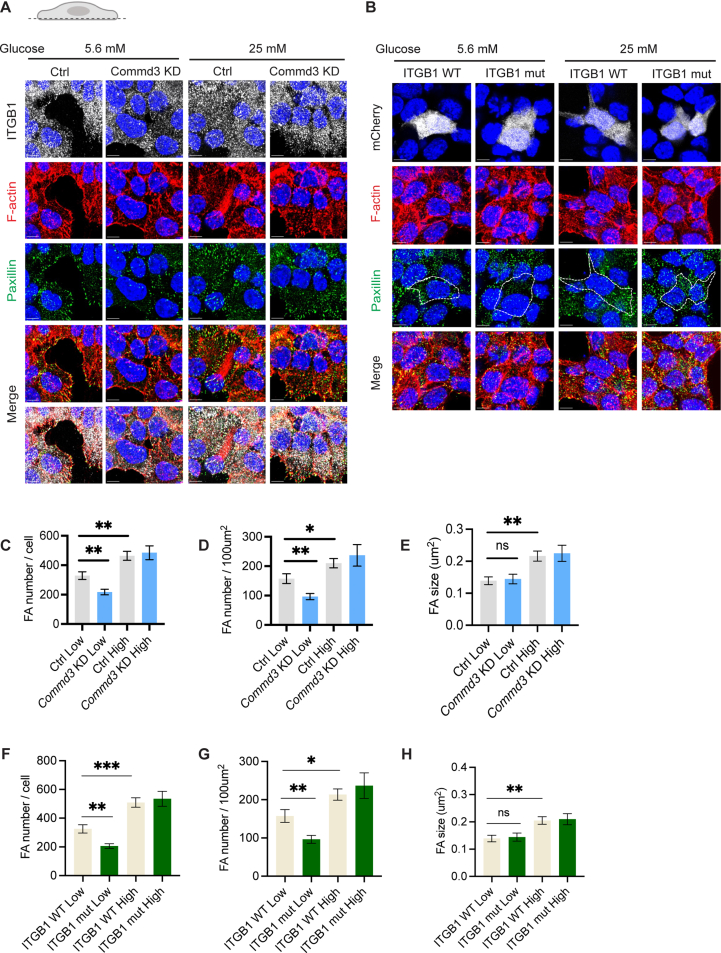
**Defective ITGB1 recycling reduces cortical focal adhesions**. (A) Representative images of ITGB1, F-actin, and ELKS localization at basal plasma membrane of control and *Commd3* KD MIN6 cells. The cells were cultured in medium containing 5.6 mM or 25 mM glucose, fixed, staining for ITGB1 (white), F-actin (red) and Paxillin (green) and imaged with confocal microscopy. (B) Representative images of F-actin and Paxillin localization at basal plasma membrane of MIN6 cells co-expressing mCherry and WT or mutant ITGB1. The cells were transfected, cultured in medium containing 5.6 mM or 25 mM glucose, fixed, stained for F-actin (red) and ELKS (green) and imaged with confocal microscopy. (C) Quantification of focal adhesion number per cell in control and Commd3 KD MIN6 cells treated as in (A). (D) Quantification of focal adhesion number per area in control and Commd3 KD MIN6 cells treated as in (A). (E) Quantification of focal adhesion size in control and Commd3 KD MIN6 cells treated as in (A). (F) Quantification of focal adhesion number per cell in MIN6 cells expressing WT or mutant ITGB1 treated as in (B). (G) Quantification of focal adhesion number per area in MIN6 cells expressing WT or mutant ITGB1 treated as in (B). (H) Quantification of focal adhesion size in MIN6 cells expressing WT or mutant ITGB1 treated as in (B). ns, no significant difference; ∗∗∗P < 0.001, ∗∗P< 0.01, ∗ P < 0.05, unpaired t test. Error bars, S.E.M.
