# Supplementary material for: Genome-wide CRISPR screen identified a role for commander complex mediated ITGB1 recycling in basal insulin secretion
Source: Mol Metab. 2022 Jul 11;63:101541. doi: 10.1016/j.molmet.2022.101541 (PMC9304790; doi:10.1016/j.molmet.2022.101541)
Supplement: Multimedia component 2 [file mmc2.docx]

| Table S1. Primers for qRT-PCR | |
| --- | --- |
| mCommd1 qpcrF | CCTGGCGAAGATGAGAGGACTT |
| mCommd1 qpcrR | TTGCTCACTGGTGATGCCACCT |
| mCommd2 qpcrF | GTCTTGAGTGGCGGCTAGATGT |
| mCommd2 qpcrR | TGAGTGCTGTGGTCGCCATTCT |
| mCommd3 qpcrF | GAGGCAGGAAAGCACCAAGT |
| mCommd3 qpcrR | ACTCCAAGCGCCAAGAAACA |
| mCommd4 qpcrF | GGTGCTGAGTTTCATCCTGTCC |
| mCommd4 qpcrR | GCTTCTCTTCGTAACAGCGGCA |
| mCommd5 qpcrF | AGCTTCCTCCAGGCTACTGT |
| mCommd5 qpcrR | GCCCCGACTACGAGTTTCAA |
| mCommd6 qpcrF | GGATGCCAAGTCGGAGGTCA |
| mCommd6 qpcrR | GCATCACTGCCACGTAAGGA |
| mCommd7 qpcrF | TCTCCGACCTGACAGAAGTGCT |
| mCommd7 qpcrR | GCCAAGACTGATCTGATTGCTGG |
| mCommd8 qpcrF | AAGGACTTTACGCGGTTTAGGAA |
| mCommd8 qpcrR | TCAGTTGCTGGAGTGTCTCTTC |
| mCommd9 qpcrF | GCCTCCTAGATAAGACGTGCTC |
| mCommd9 qpcrR | GGAAAGAGAGCCAGAATCGCCT |
| mCommd10 qpcrF | GTGTATCACAACGTGAAGCCAG |
| mCommd10 qpcrR | CTCCACGTTCTTGGCGTCTT |
| mCcdc22 qpcrF | CTGGCGAGTGTGAACCACAACA |
| mCcdc22 qpcrR | ATTCCACCGTCCTGCTCTTCAG |
| mCcdc93 qpcrF | GTCCAGAGAAGTTAGGAACCTCC |
| mCcdc93 qpcrR | GAGGCTGGTATGATTCGCTTGC |
| mVps26C qpcrF | CCACTAACTGCGCTATCACGCA |
| mVps26C qpcrR | CATCTCGTGCATAGCCTTCTGC |
| mVps29 qpcrF | TGGGCACAGATTGGTGTTGG |
| mVps29 qpcrR | GCTGGATTTTTCCTGGCACC |
| mVps35l qpcrF | CATCTCCTCGATGTGTGTGGA |
| mVps35l qpcrR | CCCGGATGGAAGCGATCTTG |
| mMafa qpcrF | GCTTCAGCAAGGAGGAGGTCAT |
| mMafa qpcrR | TCTCGCTCTCCAGAATGTGCCG |
| mUcn3 qpcrF | ACAACCAAACGCACCTCCAGATC |
| mUcn3 qpcrR | TTGTGCTGTGCCTGGGATTGGT |
| mGlut2 qpcrF | GTTGGAAGAGGAAGTCAGGGCA |
| mGlut2 qpcrR | ATCACGGAGACCTTCTGCTCAG |
| mSyt4 qpcrF | CTACAAACACGCTCACTGTGGTG |
| mSyt4 qpcrR | GGAGTGCATTTCTTAACGTGAGTC |
| mIns1 qpcrF | CGTGGCTTCTTCTACACACCCA |
| mIns1 qpcrR | TGCAGCACTGATCCACAATGCC |
| mIns2 qpcrF | CGTGGCTTCTTCTACACACCCA |
| mIns2 qpcrR | TCCAGTGCCAAGGTCTGAAGGT |
